# Counting the social, psychological, and economic costs of COVID-19 for cancer patients

**DOI:** 10.1007/s00520-022-07178-0

**Published:** 2022-06-11

**Authors:** Ann Kirby, Frances J. Drummond, Amy Lawlor, Aileen Murphy

**Affiliations:** 1 Department of Economics, Cork University Business School, University College Cork, Aras na Laoi, Western Rd, Cork, Ireland; 2 Breakthrough Cancer Research, Glenlee, Western Rd., Cork, Ireland

**Keywords:** Cancer, Covid-19, Economic, Psychological, Social

## Abstract

**Purpose:**

Cancer patients were particularly vulnerable to the adverse impacts of the COVID-19 pandemic given their reliance on the healthcare system, and their weakened immune systems. This systematic review examines the social, psychological, and economic impacts of COVID-19 on cancer patients.

**Methods:**

The systematic search, conducted in March 2021, captures the experience of COVID-19 Wave I, when the most severe restrictions were in place globally, from a patient perspective.

**Results:**

The search yielded 56 studies reporting on the economic, social, and psychological impacts of COVID-19. The economic burden associated with cancer for patients during the pandemic included direct and indirect costs with both objective (i.e. financial burden) and subjective elements (financial distress). The pandemic exasperated existing psychological strain and associated adverse outcomes including worry and fear (of COVID-19 and cancer prognosis); distress, anxiety, and depression; social isolation and loneliness. National and institutional public health guidelines to reduce COVID-19 transmission resulted in suspended cancer screening programmes, delayed diagnoses, postponed or deferred treatments, and altered treatment. These altered patients’ decision making and health-seeking behaviours.

**Conclusion:**

COVID-19 compounded the economic, social, and psychological impacts of cancer on patients owing to health system adjustments and reduction in economic activity. Identification of the impact of COVID-19 on cancer patients from a psychological, social, and economic perspective following the pandemic can inform the design of timely and appropriate interventions and supports, to deal with the backlog in cancer care and enhance recovery.

**Supplementary Information:**

The online version contains supplementary material available at 10.1007/s00520-022-07178-0.

## Introduction

Cancer is a leading cause of death globally, with almost 10 million deaths and 19.3 million incidences worldwide in 2020 [1]. This has a significant economic burden globally, estimated at $1.16 trillion in 2010 [2]. Such cost estimates capture expenditure on several types of cancer care, depending on prevalence, treatment patterns, and pharmaceutical spend. However, the economic burden of cancer extends beyond the costs of healthcare delivery. Patients and survivors also face objective costs (i.e. financial burden), arising from out-of-pocket payments. These vary depending on the public healthcare system in which they are treated, and insurance coverage. Additionally, there are subjective costs (financial distress) [3], which incorporates the psychological consequences and coping behaviours associated with the financial burden of cancer. Financial distress has adverse effects on health outcomes, collectively affecting quality of life (QoL) and well-being [3]. Recently, the COVID-19 pandemic disrupted health services globally. Cancer patients were particularly vulnerable to its adverse impacts given their reliance on the healthcare system, and their weakened immune systems.

Here, we investigate the social, psychological, and economic costs of the pandemic on cancer patients. National and institutional public health guidelines issued to protect against COVID-19 both influenced cancer care. Stay-at-home orders, social distancing, reconfigured healthcare delivery, reduced healthcare capacity, and re-distributed resources were needed to meet the demands of COVID-19. This in turn negatively impacted cancer care.

This systematic review examines the social, psychological, and economic impacts of COVID-19 on cancer patients. The systematic search, conducted in March 2021, captures the experience of COVID-19 Wave I, when the most severe restrictions were in place globally. Taking a patient perspective, the findings provide reflections on how cancer care for patients undergoing treatment was affected by the pandemic. Consideration is given to innovations arising during the pandemic and lessons learned for designing future developments and supports, which are to mitigate the social, psychological, and economic impacts associated with cancer.

## Methods

### Study selection criteria

This systematic review was conducted in accordance with the principles of conducting systematic reviews [4]. The PICOCS framework (i.e. population, intervention, comparators, outcomes, context, studies) was used to support inclusion criteria [5] (see Table 1). (There was a minor adaptation including “context” and excluding “comparator” as it was not applicable.) Studies published between January 2020 and March 2021 examining the impact of the COVID-19 pandemic on adult cancer patients undergoing treatment and survivors (2 years post-diagnosis) were examined. Inclusion and exclusion criteria are outlined in Table 1. Studies included were limited to those written in English and focused on the economic, social, and psychological implications of COVID-19 on cancer patients/survivors. Table 2

**Table 1:** Inclusion and exclusion criteria

PICOS framework	Inclusion criteria	Exclusion criteria
Population	Adult population (> 18 years old) Current cancer patients and survivors (2-years post-diagnosis)	Caregivers, nursing, and medical staff and paediatric cancer patients
Intervention	COVID-19 pandemic	-
Outcome	economic, social, and psychological implications of COVID-19 on cancer patients/survivors	-
Context	Hospital and community setting	-
Studies	Full-text articles, patient perspective, observational, cross-sectional, prospective, longitudinal, retrospective	Letters to the editor, editorials, case studies, reports, protocols, commentaries, short communications, reviews, opinions, perspectives, and discussions

**Table 2:** Search terms

Population	Intervention	Outcome			
Cancer patients	COVID-19	Economic impact	Social impact	Psychological impact	Health impact
"cancer" OR "oncology" OR "malignant" OR "tumour" OR "metastasis" OR "neoplasm"	“covid-19" OR "coronavirus" OR "2019-ncov" OR "sars-cov-2" OR "cov-19" OR "severe acute respiratory syndrome coronavirus-2" OR "pandemic"	"financial toxicity" OR "out-of-pocket" OR "productivity" OR "absenteeism" OR "unemployment" OR "cost" OR "waiting time" OR "expenses" OR "financial stress" OR "inconvenience" OR "opportunity cost" OR "income"	"well being" OR "social isolation" OR "exclusion" OR "loneliness" OR "happiness" OR "life satisfaction"	"fatigue" OR "insomnia" OR "psychological distress" OR "emotional distress" OR "anxiety" OR "depression" OR "post-traumatic stress disorder" OR "psychological"	"quality of life" OR "health-related quality of life" OR "survival" OR "mortality" OR "disease progression" OR "diagnosis" OR "screening" OR "recurrence" OR "disease stage" OR "delay" OR "support" OR "surgery" OR "treatment" OR "target therapy" OR "radiotherapy" OR "chemotherapy" OR "immunotherapy" OR "hormone therapy" OR "survivorship programme" OR "follow-up-care"

### Literature search strategy

A comprehensive search strategy was employed using a combination of free-text words and subject headings relevant to CINAHL, MEDLINE, PsycINFO, PsycArticles, and EMBASE databases and refined using Boolean operators. Searches were performed on the 31st of March 2021. Full search terms and combinations are provided in Appendices 1 and 2. The search protocol was registered (CRD42021246651).

### Data extraction and quality assessment

Data extraction is presented in tabular format to assist reporting uniformity, reproducibility, and minimising bias (provided on Table 3). The evidence was combined and summarised using a narrative synthesis. Methodological quality of studies was evaluated using the Joanna Briggs Institute critical appraisal tools (for cross-sectional, prevalence, cohort, and qualitative studies) and the Consensus on Health Economic Criteria (CHEC) list for cost analyses. Two authors performed quality assessment independently (AL and AK). If there was conflict or uncertainty, a third author was consulted. Risk of bias in a study was considered high if the “yes” score was ≤ 4; moderate if 5–6; and low risk if the score was ≥ 7 on the JBI tools. Quality review results are presented in Appendix 3.

**Table 3:** Extraction summary

Author, year Country Type of cancer	Aim	Perspective Study design Sample size Age	Data source Context and setting Study timeframe	Data collection methods Data analysis methods	Results:
Akhtar et al. (2021) India Multiple	To describe the hospitals’ experience during the first 6 months of the COVID‐19 pandemic, including the functioning of our department, clinical outcomes, the problems faced by the patients, and the lessons learned	NA Retrospective *N* = 1 institution NA	Secondary data: hospital record database Primary data: questionnaire Hospital setting April to September 2019 and 2020	• Hospital data • Patient data of difficulties encountered •Desc. stats • *χ*^2^ tests	• Outpatient consultations reduced by 62% (2019 (20,822) vs 2020 (7973)) • Inpatient admissions reduced by 58% (2019 (2840) vs 2020 (1184)) • Chemotherapy unit reduced by 56% (2019 (4896) vs 2020 (2150)) • 31% reduction in major surgeries across all sites • Higher % head and neck surgeries (absolute % are lower than those in 2019) • Average 82 patients operated by surgeons vs 119 in 2019 • Increase in telehealth and oral counterparts • High number of no-shows due to misinformation, fear of infection: • 400 patients on waiting lists for surgery did not show up Reasons: • 52% apprehension of COVID‐19 infection • 47% unawareness about the functioning of the department Difficulties faced by patients: • 58% lack of transportation • 52% apprehension of COVID‐19 infection • 17% had logistic issues • 49% inability to arrange finances • 36% had financial issues, 46% missed consultations due to financial difficulties
Baffert et al. (2021) France Multiple	To examine cancer patients’ medical management during the COVID-19 pandemic, satisfaction with care management, quality of life, and anxiety	Patient Cross-sectional, prospective, observational *N* = 189 > 18 years old	Primary data: survey Hospital setting May to June 2020	Questionnaire using: • GAD-7 scale • SF-12 scale • Patient satisfaction using a numerical scale •Desc. stats • *χ*^2^ tests • Fisher’s exact tests • Mann–Whitney *U*-test	•6% of appointments were postponed • Patients had low anxiety scores (mean: 3.2 ± 4.5) • 21 patients (11.1%) had anxiety with a GAD-7 score > 10 • 6 (3.1%) had high anxiety (GAD7 ≥ 15) • Before COVID-19, the mean physical health score was 48.5 and mean mental health score was 42.6 • After 4 weeks, physical health remained stable (mean: 46.7) but mental health decreased (mean 36.1; *p* < 0.0001) • Risk factors of anxiety included female gender and those who lived in a city apartment • Physical health score was better in patients who lived in a city apartment • Mental health score was better in patients who lived in individual houses • Factors influencing better HRQoL include retired patients, patients with children, aged > 60 years
Bakkar et al. (2020) Jordan Thyroid	To assess the impact of COVID-19 measures on thyroid cancer treatment plans	Patient and provider Retrospective *N* = 12 > 18 years old	Secondary data: medical records Primary data: anxiety scale Hospital setting 17 March to 20 May 2020	• Medical records • HAM-A scale •Desc. stats	• All surgical procedures were performed without delay as no patients had symptoms of COVID • Additional delay in receiving conventional RIA experienced by 2 patients (17%) placed them in the mild-to-moderate anxiety group according to the HAM-A scale • 50% (6/12) patients had additional extra personal cost of 1000 JOD per patient due to treatment modification
Bäuerle et al. (2021) Germany Multiple	1.To analyse individual changes in cancer patients’ mental health before and after the COVID-19 outbreak 2.To explore predictors of mental health impairment	Patient Cross-sectional *N* = 150 ≥ 18 years	Primary data: survey Hospital setting 16–30 March 2020	•Online survey using: •EQ-5D-3L •GAD-2 •PHQ-2 •Distress scale •COVID fear Likert scales •Desc. stats • Regression analysis	• Health status deteriorated since the COVID-19 outbreak (*p* = 0.004) • There was no predictor for reported change in health status • Increase in depression (*p* = 0.01), anxiety symptoms (*p* = 0.001), and distress (*p* = 0.001) • The prevalence of major depression, severe generalised anxiety, and enhanced distress all increased after the outbreak • COVID-related fear was a predictor of increased depression and generalised anxiety symptoms
Biagioli et al. (2020) Italy Multiple	To investigate the perception of self-isolation at home in patients with cancer during the COVID-19 lockdown in Italy	Patient Cross-sectional *N* = 195 ≥ 18 years	Primary data: online survey Web-based 29 March to 3 May 2020	•ISOLA scale •Desc. stats • Qualitative content analysis	• 37.3% of participants were “very or extremely” afraid of going to hospital because of the COVID-19 outbreak • 24.5% were “very or completely” afraid that their cancer care would become less important and that this would have a negative impact on their prognosis • 39.5% of patients had been in self-isolation for > 6 weeks • 60% rarely left their home • 41% of patients reported changes in their relationships with family (avoiding kisses and hugs) (*n* = 61, 31.9%) and practising social distancing (*n* = 23, 12%) • Risk factors for feeling more isolated include less education and were living without minor children • 53.8% believed they were at a higher risk of SARS-CoV-2 infection than the general population • Worried about financial difficulties and employment
Campi et al. (2020) Italy Urological	To explore urological patients willing to defer their planned surgical interventions to offer insight of patient perspective and shared decision-making	Patient Cross-sectional *N* = 2 referral centres *N* = 332 (171 scheduled for oncology surgery) Adults	Primary data: interviews Hospital setting Between 24 and 27 April 2020	• Structured telephone interview • Frailty measured by the American Society of Anaesthesiologists (ASA) score • Clinical and demographic info. from hospital databases •Desc. stats • *χ*^2^ tests • Mann–Whitney tests	• 47.9% patients would defer planned surgical intervention • 85% of them would be willing to postpone it for at least 6 months • Patients < 60 years old, frail (ASA ≥ 3), and those with underlying conditions were more willing to postpone surgery • Malignant cancer patients (33.3%) were less willing to cancel appointments compared to benign (63.4%) • 54.8% patients considered the risk COVID-19 during hospitalisation potentially more harmful than the risk of delaying surgery • Older patients were more worried about the risk of COVID-19 infection
Catania et al. (2020) Italy Lung	To better understand patients’ fears and expectations of cancer patients during the pandemic period	Patient Cross-sectional *N* = 156 Adults	Primary data: interview Hospital setting 30 April to 29 May 2020	• Structured interview • Desc. stats • Logistic regression • Fisher’s exact test • Odds ratio	• 56% reported not at all/a little worsening of QoL • 40% were afraid of COVID •55.1% in Q1 and 60.3% in Q2, respectively, reported not at all/a little worried about COVID-19 • 20% in Q1 and 14.1% in Q2 reported being quite a bit/extremely worried • 57% being more worried by their lung cancer than by COVID-19 • 17% reported more worried about COVID-19 than lung cancer • 56% reported not at all/a little worsening of QoL • Patients with comorbidities experienced fear of COVID-19 • Patients who had already received (radiotherapy or surgery) experienced more fear of COVID-19 • Females experienced more fear of COVID-19
Chaix et al. (2020) France Breast	To assess psychological distress amongst at-risk populations during the COVID-19 pandemic	Patient Cross-sectional *N* = 1771 (360 = cancer) Adults	Primary data: survey Web-based: 4 Vik chatbox 31 March to 7 April 2020	• A self-report questionnaire using: • PDI scale •Desc. stats • ANOVA •Binomial logistic regression	• The mean PDI score breast cancer = 10 • 34% (123) had psychological distress with a score ≥ 14 • Risk factors for a higher PDI score include having depression (*p* < 0.001) • Risk factors for a higher PDI include being a woman (*p* = 0.004) and unemployed (< 0.001)
Chia et al. (2021) China Multiple	To explore the emotional impact of and behavioural responses to COVID-19 amongst cancer patients and their caregivers	Patients and caregivers Qualitative *N* = 30 (16 patients, 14 caregivers) ≥ 21 years old	Primary data: semi-structured interview Hospital setting 9th and 13th of March 2020	• A semi-structured interview •Thematic analysis	• COVID-19 was the most prominent source of threat that elicited fear, worry, and perceptions of vulnerability • Threat was more pronounced in patients • Patients were concerned about personal vulnerability • Worried about impact on healthcare and prioritising cancer/treatment disruptions
Charsouei et al. (2020) Iran Breast	To investigate the perceived stress and its effect on the quality of life (QoL) and coping strategies of patients during the COVID-19 pandemic	Patient Cross-sectional *N* = 61 Adults	Primary data: survey Hospital setting 20 February to 21 May 2020	Survey using: •Perceived Stress Scale (PSS) •SF-36 questionnaire-QoL •Moos’ Coping Checklist • Desc. stats • Pearson’s correlation • ANOVA	• High scores for problem- and emotion-focused strategies were for patients with no history of radiotherapy, and attended more than 20 chemotherapy sessions • Overall perceived stress level scores were high • Higher stress was in patients with academic degree, those with a history of mastectomy, and those who attended more than 20 chemotherapy sessions • Overall QoL scores were low • Overall score of coping strategies was high • Higher stress levels mainly used problem-focused coping strategies rather than emotional-focused strategies • High scores for problem- and emotion-focused strategies were patients > 60 years old
de Joode et al. (2020) Netherlands Multiple	To assess the impact of this pandemic on oncological care	Patient Cross-sectional *N* = 5302 Adults	Primary data: survey Web-based 29 March to 18 April 2020	•Online survey •Desc. stats • *χ*^2^ tests	• 30% of all respondents experienced some consequences for their treatment or follow-up due to the pandemic • The most frequently adjusted therapies were chemotherapy and immunotherapy • The most frequently reported consequence was the conversion to consultation by phone or video (52%) • 39 of 250 patients’ treatments were postponed • 279 of 2391 patients awaiting and under treatment • 49 of 250 patients experienced treatment changes (adjustment, delay, and discontinuation of treatment), while 480 of 2391 were awaiting and under treatment • Most patients with curable disease continued their treatment unchanged • Incurable patient’s treatment was more frequently postponed • 47% of respondents were (very) concerned to be infected with COVID-19 • Among patients with delay and discontinuation of treatment, 55% and 62% of patients were concerned, respectively • Among patients who did not experience consequences yet, 24% of patients were (very) concerned about potential consequences for their treatment or follow-up • Patients with cured disease or follow-up, 87% and 83% of patients were not/slightly concerned, respectively. Incurable patients were more concerned of COVID-19 infection • Patients who were under treatment were more often (very) concerned to be infected than patients in follow-up • 19% of patients were reluctant to contact their hospitals during the pandemic
Deshmukh et al. (2020) India Multiple	1. To critically assess and quantify the response of a small single specialty cancer centre to the pandemic 2. To analyse the impact of a pandemic of this magnitude on cancer patients’ treatment	NA Retrospective *N* = 1 institution *N* = 3 departments NA	Secondary data: hospital records Hospital setting Pre-COVID: 22 March to 31 May 2019, 2018, 2017 Lockdown: 22 March to 31 May 2020	•Hospital data •Desc. stats	• During the lockdown period: 28 patients underwent surgery, 469 underwent CT, and 56 patients underwent RT • In 2019: 929 patients underwent surgery, 7355 underwent CT, and 1037 underwent RT • Number of surgeries in 2020: 16 head and neck surgeries and 12 other malignancies • Number of surgeries in 2019: 366 head and neck surgeries and 563 other malignancies • Average number of patients treated per week in surgical department in the lockdown period dropped to 5.2 (from a range of 14 to 21 for the other 3 years) • CT and RT average number remained stable • Financial pressure of increased hospital length and COVID-19 testing
Elran-Barak and Mozeikov (2020) Israel Multiple	1. To examine how the lockdown measures impacted the self-rated health (SRH), health behaviours, and loneliness of people with chronic illnesses 2. Determine socio-demographic or medical-related factors linked to a decline in SRH	Patient cross-sectional *N* = 315 (64 cancer patients) > 18 years old	Primary data: online survey Web-based 20 to 22 April 2020	Online survey using: • SF-36, medical outcomes • The Challenges to Illness Management Scale • The Revised UCLA Loneliness Scale • Desc. stats • *T*-tests • Ordinal logistic regression •ANOVA • *χ*^2^ tests	• 47.2% reported a decline in their physical health • Changes in physical health for cancer/autoimmune by an average of 3.53 (SD = 0.78) • Changes in general health by an average of 3.08 (SD = 0.82) • 50.5% reported a decline in mental health during the first month of the COVID-19 outbreak • Sense of loneliness was statistically significant among all patients (*T* = 12.76, *p* < 0.001) • Feelings of loneliness amongst cancer/autoimmune group scored an average of 5.66 (SD = 2.15) • Changes in mental health scored an average of 3.48 (SD = 0.77) Decline in general SRH was predicted by: • Female gender (*p* = 0.016), • Lack of higher education (*p* = 0.015) • Crowded housing conditions (*p* = 0.001) • Illness duration (*p* = 0.010)
Erdem and Karaman (2020) Turkey Multiple	1. To assess the knowledge, perceptions, and attitude of patients with cancer towards the COVID-19 pandemic 2. To measure the effect of COVID-19 on cancer patients’ ongoing treatments	Patient Prospective cross-sectional *N* = 300 19–92 years old	Primary data: questionnaire survey Hospital setting 1 to 30 April 2020	• Survey questionnaire •Desc. stats •Kolmogorov–Smirnov test •Shapiro–Wilk test • *T*-test • *χ*^2^ tests • Fisher–Freeman–Halton Exact test •Fisher’s exact	• 98% had no delay for current cancer treatments or follow-up appointments • 52.3% using nutritional supplements • One-third of patients were afraid to leave their house • One-third of patients left their house only for the hospital during this period • 96% prefer not to use public transport due to risk of COVID-19 • One-third of patients never left their house • 97% of patients did not accept visitors to their houses • Two-thirds of patients went out with a mask • 97.3% were washing their hands more often than usual • Patients over 65 years old were most prone to stay at home • Male patients were more likely to leave their home • Patients with stage 1 cancer tend to stay at home, while patients with stage 4 cancer were more likely to leave their houses for hospital visits at a higher ratio • Patients with less than high school degree were more prone to stay at home • Higher educational status was associated with better knowledge of routes COVID transmission
Fox et al. (2021) UK Multiple	To determine if there were gender differences in participants’ concerns about taking part in cancer research and anxiety levels of cancer patients during the pandemic	Patients Cross-sectional *N* = 93 ≥ 18 years	Primary data: survey Web-based 5th and 19th of June 2020	• Online survey using: • GAD-7 • Desc. stats • Kruskal–Wallis tests • Linear regression • *χ*^2^ tests • *T*-tests	•Higher concerns of risk include previously received cancer treatment and varied by type of cancer • Females were less likely to participate, or would not participate, in research due to COVID-19 • Females had a significantly higher score for “Total concerns” category (*p*-0.004) and anxiety levels (*p* < 0.001) • Age and travel for treatment were concerns for COVID-19 risk
Frey et al. (2021) USA Ovarian	To assess coping strategies employed by women with ovarian cancer during the COVID-19 pandemic	Survivors Cross-sectional *N* = 408 NA	Primary data: survey Secondary data: quality of life and treatment interruptions 30 March to 13 April 2020	• Online survey using: • Brief COPE framework •Desc. stats	•33.9% (113) reported a delay in some component of their cancer care • 8.6% reported that their treatment was postponed •27.6% reported surgery was delayed Adaptive coping strategies: • Emotional support (39%) • Self-care (36.3%) • Hobbies (34.1%) • Humour (1.7%) • Planning (21.3%) • Positive reframing (13.2%) • Religion (12.3%) • Instrumental support (9.3%) • Acceptance (3.9%) Dysfunctional strategies: • Substance use (4.7%) • Venting (2.9%) • Behavioural disengagement (1.5%) • Self-distraction (27.2%) • Self-blame (0.5%)
Frey et al. (2020) USA Ovarian	1. To evaluate the quality of life of women with ovarian cancer during the coronavirus disease 2019 pandemic 2. Evaluate the effects of the pandemic on cancer-directed treatment	Patients and survivors Cross-sectional *N* = 555 20–85 years old	Primary data: survey Web-based 30 March to 13 April 2020	• Online survey using: • HADS scale • Cancer Worry Scale • *T*-test • ANOVA • Mann–Whitney *U* test • Kruskal–Wallis test • Linear regression	• 16.6% worried about QoL and wellness • 33% experienced a delay in cancer care • 26.3% scheduled for surgery experienced a delay • 24% had a delayed physician appointment • 25% used telemedicine for gynaecologic oncology care • Adaption of telemedicine was associated with higher levels of cancer worry • 26.9% worried about access to care • 58% worried about COVID-19 infection • 57% worried about cancer recurrence • 89% reported significant cancer worry • Younger age, presumed immunocompromised, and delay in care were associated with a significant increase in cancer worry, anxiety, and depression • 51.4% (285) borderline or abnormal anxiety, and 26.5% (147) borderline or abnormal depression • Age < 65 years was associated with higher levels of worry • 10% were concerned about social isolation • 24.3% were concerned about the financial implications of COVID-19
Gebbia et al. (2020) Italy Multiple	To investigate if instant messaging systems are useful to oncologists to care for patients with cancer and to mitigate patient anxieties and fears during the COVID-19 outbreak?	Patient Observational *N* = 446 ≥ 18 years	Secondary data: patient queries Hospital setting 8 to 22 March 2020	• Spontaneous patient queries were collected through a chat text •Desc. stats •Sentimental analysis • *χ*^2^ tests	• 37% asked if they can postpone their appointments • 198 follow-up visits were delayed after queries or independent oncologist suggestions • 5 patients asked for a delay in adjuvant radiotherapy • A majority of delays were in patients with breast, colon, or prostate cancer with programmed follow-up visit • Majority of queries came from the most prevalent cancers (breast, lung, colon, prostate) • Fear was the most common emotion • Fear, anger, and sadness most dominant negative emotions • 57% showed negative emotion • 43% showed positive emotions • 50% felt trust • Patients > 75 years old more commonly requested visit/treatment delays
Gheorghe et al. (2020) Romania Multiple	1. To describe the level of knowledge, attitude, and practices (KAP) related to COVID-19 among cancer patients 2. To evaluate the effectiveness of pandemic response measures	Patient Cross-sectional *N* = 1585 patients, *N* = 7 hospitals Adults	Primary data: questionnaire survey Hospital setting 27 April to 15 May 2020	• Questionnaire • Desc. stats • Regression analysis	•Better knowledge of COVID-19 was associated with patients aged between 40 and 54 years, higher education programme, female, urban areas, profession of mental labour, higher income • 68% considered cancer as an additional risk for infection with SARS-CoV-2 • 27.8% would rather not vaccinate • 8.8% believed risk of infection justifies delaying/stopping oncological treatment • 55.5% declared being compliant with COVID protective measures • Distress of risk of COVID-19 was higher, compared to influenza virus • 32.6% were “very worried” about getting infected with the coronavirus or developing COVID-19 • 35.9% were “somewhat worried” • 11.6% feared COVID-19 infection more than cancer progression • 61.8% feared of both events in equally • Those with low income, low socioeconomic status, and higher education were more worried about COVID-19 • Very few patients would rather stop their treatment
Ghosh et al. (2020) India Multiple	To assess the mindset of patients about continuation of anticancer systemic therapy during this pandemic	Patient Prospective observational *N* = 302 patients ≥ 18 years	Primary data: survey Hospital setting 1 to 10 April 2020	•Questionnaire-based survey •Desc. stats • *χ*^2^ tests • *T*-tests • Fisher’s exact test • Pearson’s correlation	• 203 (68%) patients wanted to continue chemotherapy, 40 (13%) wanted to defer, and 56 did not know (19%) • No correlation of intent of treatment with chemotherapy willingness • Knowledge of COVID-19 was almost evenly distributed among well informed, moderately informed, and minimally informed Worried about COVID-19 infection: • Very much, 58 (19%) • Moderate, 126 (42%) • Minimal, 118 (39%) • Worry about disease progression was more common in palliative patients • Fear of COVID-19 over cancer directly correlated with higher knowledge about immunosuppression • Patients were predominantly bothered about deferring chemotherapy (45), visiting hospitals (50), both (100), or about cancer progression (104) if therapy deferred
Goenka et al. (2020) USA Not specified	Review implementation of telemedicine 1. Patient access to care 2. Billing implications	Provider Observational *N* = 1 institution 22–93 years old	Secondary data: hospital data Hospital setting 1 January to 1 May 2020	•Telemedicine platform • Desc. stats • Logistic regression	• 2997 billable evaluation and management encounters occurred • 35% decrease in billable activity • In-person visits decreased from 100 to 21% • 60% were 2-way audio–video • 40% by telephone only • Older patient age was less likely to have 2-way audio–video encounters • The financial impact of the transition to telehealth must be considered including the cost of telehealth implementation and maintenance, the number of second opinion consults, the difference in reimbursement, cost savings to patients (direct and indirect), and cost savings from care coordination
Greco et al. (2020) Italy Prostate renal	To investigate the impact of postponement of surgeries due to the COVID-19 on the on HRQOL of uro-oncologic patients	Patients Cross-sectional *N* = 50 Adults	Primary data: survey Hospital setting 1 March to 26 April 2020	• SF-36 questionnaire • Desc. stats	• 86% reported normal physical functioning but loss of energy • Most patients reported change in emotional functioning: increase in anxiety and depression • All patients perceived a reduction in general health condition
Gultekin et al. (2020) Europe Gynaecological	To capture the patient perceptions of the COVID-19 implications and the worldwide imposed treatment modification	Patients Prospective 16 EU countries > 18 years old	Primary data: survey Hospital setting 1 to 31 May 2020	• COVID-19-related questionnaire • HADs scale •Desc. stats • Logistic regression	• 71% were concerned about cancer progression if their treatment/follow-up was cancelled/postponed • 64% had their care continued as planned • 5.1% said that their surgery was delayed • 7% said that their imaging was cancelled or disrupted • 2.8% reported a delay in their chemotherapy or radiotherapy (0.5%) appointments • 12.8% reported follow-up was postponed or delayed • Mean HADS Anxiety and Depression Scores were 8.8 and 8.1 respectively • 35.3% had an abnormal HADS Anxiety score • 30.6% had an abnormal depression score • Treatment modifications of care and concerns of care were predictors of patients’ anxiety • 7.4% patients reported not attending their treatment/follow-up appointments due to fear of COVID-19 infection • 17.5% were more afraid of COVID-19 than their pre-existing malignant diagnosis • 53.1% expressed their fear of contracting COVID-19 from the hospital • Those aged 70 years or older were more afraid of COVID-19 compared to cancer (*p* < 0.001)
Han et al. (2020) China Multiple	To assess the psychological status and symptoms of cancer survivors and family members compared to Chinese norms	Survivors and family members Longitudinal *N* = 111 33–75 years old	Primary data: survey Web-based T1: 14 to 24 February T2:1 to 10 April T3: 15 to 25 May 2020	• Online questionnaire using: • Symptom checklist 90 (SCL-90) •Desc. stats • MANOVA • *T*-test	• Survivors’ mean total score of the SCL-90 for T1: 172.05 (SD = 13.30), T2: 155.91 (SD = 12.18), T3: 142.75 (SD = 11.56) • Survivors’ SCL-90 score was significantly higher than that of their family members • Family members had significantly higher SCL-90 scores than Chinese norms (T = 3.03, *p* = 0.001) • Somatisation, depression, anxiety, and phobic anxiety scored the highest on the SCL-90 scale for survivors
Hill et al. (2021) USA Ovarian	1. To examine the role of intolerance of uncertainty (IU) in psychological distress (PD) among women with ovarian cancer 2. Fear of COVID-19	Patient Cross-sectional *N* = 100 ≥ 18 years	Primary data: survey Web-based 1 July and 30 October 2020	• Online survey using: • Intolerance of Uncertainty Scale • Fear of COVID-19 Scale (FCS) • Depression Anxiety Stress Scales (DASS-21) • Desc. stats • Linear regression	• Depression and anxiety models were significant • Higher levels of IU were associated with depressive symptoms • Lockdown status of the geographic area (red or yellow status) was associated with increased depressive symptoms • Fear of COVID was not significant for depressive symptoms • Fear of COVID was the strongest predictor for anxiety • Fear of COVID and Intolerance of Uncertainty were strongly correlated • Stress model was significant with IU the strongest predictor
Islam et al. (2020) USA Not specified	1. To evaluate COVID-19-related preventative measures among cancer survivors 2. To examine behaviours related to cancelling or postponing activities, specifically doctors’ appointments	Survivors Cross-sectional *N* = 854 ≥ 18 years	Secondary data: from the US COVID-19 Household Impact Survey. Primary data: interview Community based Week 1 (April 20–26, 2020), week 2 (May 4–10, 2020), week 3 (May 30–June 8, 2020)	• Sample from the national household survey •Telephone and face-to-face interviews •Demographic details from the 2020 Current Population Survey • COVID-19 deaths were obtained from USA facts •Desc. stats •Regression analysis	• Between April and May, the proportion of cancer survivors that cancelled a doctor or dentist's appointment increased from 35 to 52% and 36 to 49%, respectively • Preventative behaviours amongst cancer survivors compared to the general population were statistically significantly more likely to wash or sanitize their hands, social distance, wear a face mask, avoid public or crowded places, avoid some or all restaurants, avoid contact with high-risk people, and cancel pleasure, social, or recreational activities • Cancer survivors were also more likely to cancel doctor appointment or postpone a dentist or other appointment compared to the general population •Widowed/divorced/separated were less likely to cancel doctor’s appointments compared with those who were married • Those aged 18 to 29 were more likely to cancel a doctor’s appointment compared with those aged 60 years and above • H-Black survivors are less likely to cancel a doctor’s appointment when compared with NH-White survivors •Female and co-morbid survivors were more likely to cancel appointments
Jeppesen et al. (2020) Denmark Multiple	To investigate patient's quality of life (QoL), emotional functioning, and concerns about COVID-19	Patient Cross-sectional *N* = 4571 > 18 years old	Primary data; survey Hospital setting 15 to 29 May 2020	• Online survey using: • EORTC QLQ-C30 instrument-Health-related quality of life •Desc. stats • *χ*^2^ tests • Linear regression model	• 9% of all patients with cancer had refrained from consulting a doctor or the hospital due to fear of COVID-19 infection • 80% were concerned about contracting COVID-19 • Female, comorbid, conditions, incurable cancer, or receiving medical cancer treatment was associated with higher concern of contracting COVID-19 • Concerns of contracting COVID-19 infection were correlated with lower QoL and the emotional functioning scores • Higher quality of life was correlated with older age, not living alone, employed, fewer comorbidities, and not receiving treatment within the last 2 months • Patients with brain tumours, and endometrial/cervical/vulva and thoracic cancers had lower quality of life score • Better emotional functioning was correlated with male gender, older age, fewer comorbid conditions, and not receiving treatment within the last 2 months
Juanjuan et al. (2020) China Breast	To evaluate patient-reported outcome in patients with breast cancer and survivors	Patients and survivors Cross-sectional *N* = 658 *N* = 12 cancer centres NA	Primary data; survey Hospital setting 16 to 19 February 2020	• Online survey using: • GAD-7 scale • PHQ-9 scale • Insomnia Severity Index (ISI) • Impact of Events Scale-Revised (IES-R) • Desc. stats • Wilcoxon rank-sum test • Kruskal–Wallis test • Logistic regression	• 46.2% of patients had to discontinue or modify their planned necessary anticancer treatments • Poor general condition, treatment discontinuation, and metastatic breast cancer were more likely to experience severe symptoms of anxiety, depression, insomnia, and distress • Mean score for GAD-7 = 6.01 (SD = 5.35) •34.0%, 13.3%, and 8.9% patients categorized into the mild, moderate, and severe anxiety, respectively • Mean score for PHQ-9 = 5.80 (SD = 5.66) • 25.2%, 12.8%, and 9.3% patients who reported mild, moderate, and severe depression, respectively • Mean score for ISI = 8.66 (SD = 6.29) • 36.2%, 12.9%, and 4.0% patients, respectively, who reported mild, moderate, and severe insomnia • IES-R total = 28.17 (SD = 18.23) • 30.7%, 31.5%, and 20.8% patients who described mild, moderate, and severe distress symptoms
Kamposioras et al. (2020) England Colorectal	1. To investigate the perception of service changes imposed by COVID-19 2. To identify the determinants of anxiety in patients with colorectal cancer	Patient Cross-sectional *N* = 143 ≥ 18 years	Primary data: survey Hospital setting 18 May to 1 July 2020	• Survey using: • GAD-7 scale •Desc. stats • *χ*^2^ tests • Fisher exact test • Logistic regression	• 78% participants had telephone consultation (83% met needs) • 40% had radiologic scan results discussed over the phone (96% met needs) • 90% felt safe visiting their hospital • 10% participants who had their assessment scans delayed or cancelled • 18% participants were considered to have anxiety (score ≥ 5) • 5.5% scoring for moderate or severe anxiety • 80% were concerned about COVID-19 infection • 87% denied that they were more concerned about COVID-19 than their cancer • Patients concerned about COVID-19 infection, effects on mental health, and cancer care were most likely to have anxiety • 97% reported that they were well-supported by their families and friends
Kim et al. (2021) South Korea Breast	Explore whether COVID-19–related treatment changes (delays, cancellations, changes) influenced fear of cancer recurrence, anxiety, and depression in breast cancer patients	Patient Cross-sectional *N* = 154 ≥ 20 years	Primary data: survey Web-based April to June 2020	• Online survey using: • Fear of Cancer Recurrence Inventory (K-FCRI) • HAD scale • Desc. stats • *χ*^2^ tests • Fisher’s exact test • *T*-test • ANOVA	• 18.8% had experienced COVID-19-related treatment changes • 24.1% had treatment plan changes • 62.1% experienced delays • Follow-up or tests were the most frequently delayed care • 31% treatments were cancelled • Fear of cancer recurrence was higher in patients receiving radiation therapy • Depression was more severe in patients receiving chemotherapy • 15% had moderate to severe levels of anxiety • 24.7% had moderate to severe levels of depression • Changes of the treatment plan had a significant correlation with depression (*t* = 2.000, *p* = .047) • Fear of cancer recurrence was high (mean score, 84.31 (SD 24.23) • 49.2% felt anxious about getting COVID-19 infection when in hospital for treatment • Participants who experienced treatment changes were younger, were not married, had no children, or lived more than 2 h from the hospital. Fear of cancer recurrence was significantly higher among unmarried and no children • Anxiety was more severe in lower income households • Depression was more severe in those who were unmarried, had no children, had a lower income • 6.21% felt an economic burden as testing for COVID-19 • Anxiety was more severe in those who reported a financial burden
Košir et al. (2020) Worldwide Not specified	1. To gather evidence of the impact of COVID-19 on AYA cancer patients’ and survivors’ psychological well-being and cancer care 2. To understand where they received the information about the pandemic and how satisfied they were with the resources on COVID-19	Patients and survivors Mixed methods, cross-sectional *N* = 177 18–39 years old	Primary data: survey Web-based 6 April to 11 May 2020	• Online survey using: • PHQ-4- depression and anxiety •Desc. stats •Qualitative content analysis	• 45% reported an impact on their cancer treatment. (postponed or cancelled, virtual care, reduced access to medicines) • Individuals undergoing treatment (or within the last 6 months) reported higher levels of psychological distress on average • 62% of respondents reported feeling more anxious than they did before the pandemic • 52% reported feeling more isolated than before the pandemic. Missed social interactions, low mood • Most common concern was contracting COVID-19 • 56% reported wanting more information about how to cope with the pandemic
Leach, et al. (2021) USA Breast, Male other and female other	To examine cancer survivor worries about treatment, infection, and finances early in the US COVID-19 pandemic	Survivors Cross-sectional *N* = 972 quantitative, *N* = 659 for qualitative question ≥ 18 years	Primary data; survey Web-based 25 March to 8 April 2020	• Online survey •Desc. stats • Logistic regression analysis • Thematic analysis	• Female other cancers and male survivors were more worried about treatment disruption • Female breast cancer and female other cancers were more worried about health impacts • 77% were worried about risk COVID-19 infection • Longer time since last treatment was associated with less worry • Delayed appointments due to fear of getting COVID sometimes led to greater anxiety, worry about recurrence, and health complications • Fear of rationing of care as seen as not eligible for COVID treatment • Age, education, marital status, and race/ethnicity were not associated with treatment worry or COVID-19 worry • Patients reported loneliness and feelings of being isolated due to social distancing • Non-Hispanic white, married, more educated, and older were associated with less financial worry • Concerns included employment, economic downturn, inability to pay for expensive healthcare costs (refill prescriptions and insurance deductibles)
Lou, et al. (2020) USA Multiple	To compare concerns about COVID-19 among individuals undergoing cancer treatment to those with a history of cancer not currently receiving therapy and to those without a cancer history	Patient Cross-sectional *N* = 543 ≥ 18 years	Primary data: survey Web-based 3 to 11 April 2020	• An online survey using: • GAD-7 scale • PHQ-8 scale • *χ*^2^ tests • ANOVA • Fisher’s exact tests • T-test	• 20% reported changes in care • 50.8% metastatic patients reported COVID-19 had negatively affected their cancer care (31% non-metastatic) • Chemotherapy delays most common • More than 90% in active treatment feared COVID infection • 40% expressed concerns about effects on their cancer-directed therapy plans • Higher levels of family distress • Anxiety and depression did not differ significantly between patients actively being treated for cancer vs no history of cancer
Mahl et al. (2020) Brazil Head and neck	To evaluate delays in care for patients with head and neck cancer (HNC) in post-treatment follow-up or palliative care during the COVID-19 pandemic; i.e. self-perception of anxiety or sadness, fear of COVID-19 infection, cancer-related complications during social isolation, self-medication, diagnosis of COVID-19, and death between patients with and without delayed cancer care	Patient cross-sectional *N* = 1 institution *N* = 31 patients NA	Primary data: interview Secondary data: medical records Hospital setting 1 January to 30 July 2020	• Telephone interviews • Desc. stats • Mann–Whitney *U* test • Fisher’s exact test	• 58.1% had delayed cancer care (18/31) • No report of telemedicine use • Increase in self-medicating in patients who had delayed treatment • Fear of COVID infection: 41.9% (*n* = 13) • Feelings of anxiety: 71.0% (*n* = 22) • Sadness 45.2% (*n* = 14)
Mari et al. (2020) Italy Multiple	1. To determine the extent the pandemic has had on surgical procedures for cancer, benign, and emergency cases 2. Perform a cost analysis	Provider Retrospective *N* = 4 hospitals NA	Secondary data: hospital data Hospital setting March, April and May 2019 and 2020	• Surgical volumes from surgical registries from 4 different hospitals •Desc. stats • Cost analysis of hospital revenue	• 60.1% reduction in cancer surgeries from 403 to 161 • 81.6% reduction in overall surgeries • 57.3% reduction in state funding for cancer surgical procedures performed. Reimbursement falling from €2.3 million to €967,333 between 2019 and 2020
Massicotte et al. (2020) Canada Breast	To examine stressors related to the ongoing COVID-19 pandemic and their relationships with psychological symptoms (i.e. anxiety, depression, insomnia, and fear of cancer recurrence (FCR) in breast cancer patients undergoing cancer treatments)	Patient Cross-sectional *N* = 36 18–80 years old	Primary data: questionnaire Hospital setting 28 April to 29 May 2020	• Questionnaire using: • Insomnia Severity Index (ISI) • HAD scale • Fear of Cancer Recurrence Inventory (FCRI). • COVID-19 Stressors Questionnaire • Desc. stats • Kendall’s Tau • Pearson’s correlation	• Stressors that were associated with the postponement or cancellation of cancer treatment, changes in cancer care trajectory, and postponement of medical tests • 63.9% of participants experienced at least one stressor related to the COVID-19 pandemic (one: 27.8%, two: 22.2%, three: 11.1%) • Higher levels of concerns related to the experienced stressors were significantly correlated with higher levels of anxiety, depressive symptoms, insomnia, and fear of cancer recurrence • A higher number of stressors experienced was significantly associated with greater levels of anxiety, depression and insomnia, but not fear of recurrence
Merz et al. (2020) Italy Breast	To assess how breast cancer survivors perceived electronic medical record–assisted telephone follow-up	Survivors Prospective *N* = 137 34–89 years old	Primary data: survey Web-based 9 March and 2 June 2020	• Online survey •Desc. stats • Pearson’s test • Fisher’s exact test • Mann–Whitney U test • χ^2^tests	• 80.3% were satisfied with E-TFU compared to a standard FU visit • 89.8% were satisfied with the duration of the phone call • 43.8% would like to have electronic medical record assisted telephone follow-up in the future • Nearly 64% suffered from COVID-19–related anxiety about their health • Low educational level was correlated with higher COVID-19–related anxiety
Miaskowski et al. (2020) USA Multiple	To evaluate for differences in demographic and clinical characteristics, levels of social isolation and loneliness, and the occurrence and severity of common symptoms between oncology patients with low vs. high levels of COVID-19 and cancer-related stress	Patient Cross-sectional *N* = 187 ≥ 18 years	Primary data; survey Web-based 27 May to 10 July, 2020	• Online survey using: • Karnofsky Performance Status scale • Self-Administered Comorbidity Questionnaire (SCQ) • IES-R scale • Perceived Stress Scale • Connor Davidson Resilience Scale • COST scale • The Los Angeles Loneliness Scale • Social Isolation Scale •CES-D scale • Spielberger State-Trait Anxiety Inventories • General Sleep Disturbance Scale (GSDS) • Lee Fatigue Scale • Attentional Function Index • Brief pain inventory •Desc. stats • *t*-tests • *χ*^2^ tests • Mann–Whitney *U* tests	• 31.6% were categorized in the Stressed group (score of > 24) • Perceived the Stressed group’s Impact score equates with probable PTSD • Stressed group, patients reported occurrence for depression (71.2%), anxiety (78.0%), sleep disturbance (78.0%), evening fatigue (55.9%), cognitive impairment (91.5%), and pain (75.9%) • The stressed group had lower score for financial toxicity (greater financial concerns)
Mitra et al. (2020) India Multiple	To study the challenges faced by cancer patients in India during the COVID-19 pandemic	Patient Cross-sectional *N* = 36 ≥ 18 years	Primary data: survey Web-based 1 to 15 May 2020	• Online questionnaire • Self-grading anxiety levels and the reason for their anxiety • Desc. stats	• 94.4% reported lack of peer group support services and psychological counselling sessions • 41.7% reported problems with slot availability for teleconsultation, while 33% had network issues • 22% reported deferral of radiotherapy dates and long waiting hours beyond appointment time • 88.9% reported delay of advice of the nutritionist • 13.8% deferral of survey, 19.5% of tumour board deferral • 76.6% reported restrictions • 91.7% reported an increase in anxiety • 8.3% reported their anxiety remained the same • 91.7% feared infection with COVID-19 was the reason for increased anxiety • 86% reported fear of disease progression increased anxiety • 55.6% reported treatment not being optimum as the reason for their increased anxiety • 27.8% reported increased anxiety due to fear of death • 22.2% reported fear of losing jobs and financial crisis for the family members as the cause of their increased anxiety
Ng. K et al. (2020) Singapore Not specified	1. To evaluate the psychological effects of COVID-19 on patients with cancer, their caregivers, and health care workers (HCWs) 2. To evaluate the prevalence of burnout among HCWs	Patients and caregivers Cross-sectional *N* = 624 patients (408 care givers, 421 HCWs) > 21 years old	Primary data: survey Hospital setting 6 to 22 April 2020	•Questionnaire survey using: • GAD-7 scale • Self-reported fears related to COVID-19 • Maslach Burnout Inventory • Desc. stats • *χ*^2^ tests • Logistic regression	• 66% of patients reported a high level of fear from COVID-19 • The greatest concern of patients was the wide community spread of COVID-19 • 19.1% of patients had anxiety (score ≥ 10) • Fear was the most common emotion, followed by anxiety • Anxiety was significantly higher in patients married, education lower than tertiary level • Patients that were non-Chinese and married had a higher level of COVID-19 fears
Papautsky and Hamlish (2021) USA Breast	1. To examine the impact of COVID-19 on health-related worry of breast cancer survivors (worry associated with: delays in cancer care, risk to general health, and risk of COVID-19) 2. To examine the role of the relationship with their cancer care team (trust, communication, planning) in models of vulnerability and worry	Survivors Cross-sectional *N* = 633 Adults	Primary data: questionnaire Web-based 2 April to 14 May 2020	•Questionnaire • Desc. stats • Pearson correlations • *T*-tests •ANCOVAs	• Patients in active treatment, immunocompromised, and experiencing delays treatment were more worried about their cancer • Trust negatively correlated with worry • Significant positive correlations between communication and trust and negative correlations between trust and cancer-related worry
Parikh et al. (2020) USA Breast	To perform a cost analysis on the transitions to telemedicine in a radiation oncology department	Payer and patient Descriptive study *N* = 1 patient NA	Primary data: interviews and surveys of personnel Hospital setting Using a patient undergoing 28-fraction treatment course, exact timeframe not specified	• Process maps were created for traditional in-person and telemedicine-based workflow processes • Interviews with personnel to obtain time spent and resource • Costs from the department’s financial officer • Time-driven activity-based costing	• Majority of consultations, follow-up visits, and on-treatment visits were converted to telemedicine •Telemedicine reduced provider costs $586 compared with traditional workflow • Patients saved $170 per treatment course
Philip et al. (2020) UK Lung	To identify and explore the concerns of people with long-term respiratory conditions in the UK regarding the impact of the COVID-19 pandemic and how these concerns were affecting them	Patients Qualitative *N* = 7039, 42 lung cancer patients NA	Secondary data: online survey Web-based 1st to 8th of April 2020	• Data from an online survey by the Asthma UK and British Lung Foundation (AUK-BLF) •Thematic analysis	• Four key themes were identified, which were concerns about (1) vulnerability to COVID-19 (most dominant theme), (2) anticipated experience of contracting COVID-19, (3) pervasive uncertainty, (4) inadequate national response 2. Mental health impacts: anxiety and fear
Pigozzi et al. (2021) Italy Multiple	To evaluate the psychological status of patients before and during the pandemic	Patient Prospective *N* = 474 20–97 years old	Primary data: survey Secondary: medical records Web-based 27 April to 7 June 2020	•Questionnaire using: •Emotional Vulnerability Index (EVI) •Desc. stats • *χ*^2^ tests	•Chemotherapy patients reported high vulnerability • Breast cancer patients felt the most vulnerable (56%) • Prostate cancer and stomach cancer patients felt the least vulnerable. Only 28% of prostate cancer patients and 27% stomach cancer patients Pre-emergency period: • Low level of emotional distress • 39% were not able to cope with their cancers During pandemic period: • 216 (47%) reported they remained the feeling of low vulnerability • 41 (9%) increased vulnerability • 10 (2%) decreased vulnerability • 196 (42%) remained feeling of high vulnerability • 90% of respondents reported strong family support •Higher vulnerability was found in females and age ≤ 65 years old
Rajan et al. (2021) India Not specified	1. To assess the impact of COVID-19 on cancer healthcare from the patient perspective 2. Analyse any adverse effects of the pandemic	Patient Cross‐sectional *N* = 310 patients > 18 years old	Primary data: questionnaire Hospital setting 19 June to 7 August 2020	• Questionnaire •Desc. stats •Binary logistic regression	• Access to care had a statistically significant difference of (34.23 ± 15.38) *p* < 0.001 • Education below a secondary school level and illiterate patients had more problems in healthcare access • 21% of patients were denied treatment • Anxiety domain had a statistically significant impact score of (24.95 ± 14.01), *p* < 0.001 • 62% had anxiety • Married participants had greater levels of anxiety • Depression domain had a statistically significant impact score of (31.24 ± 19.79), *p* < 0.001 • Two-thirds of patients had felt their life has become meaningless, and they could not experience a positive feeling in life • 81% of patients felt sad and helpless • Stress domain had the least effect with a score of (20.54 ± 13.53), *p* < 0.001 • Those earning INR < 35 K annually had more stress and depression •25% of patients suffered from insomnia • Financial status had the greatest statistically significant impact score of (59.68 ± 16.52), *p* < 0.001 •52% of patients experienced financial difficulties reporting a loss of their family earnings • Married participants had greater levels of financial impact • 45% of patients could not arrange finances and social support from their relatives or friends • 81% of participants do not have treatment covered under any government health scheme or insurance • Those earning INR < 35 K had less financial impact than those earning more as they were supported by government funds for their cancer treatment • Age 31–50 years, males, married, daily wagers, having a senior secondary level of education, and income of INR 35 K–100 K were most financially impacted • COVID-19 had the greatest impact on those with income INR < 35 K and 35 K–100 K, married, rural residence
Shinan-Altman et al. (2020) Israel Breast	To explore factors associated with health services utilization among breast cancer patients during the coronavirus disease (COVID-19) outbreak	Patient Cross-sectional *N* = 151 patients > 18 years old	Primary data: survey Hospital setting April 5 to April 12, 2020	Online survey: • Anxiety The Brief Symptom Inventory • Multidimensional Scale of Perceived Social Support • Susceptibility to COVID-19 5-point Likert-type scale • Sense of mastery 7-point Likert scale • Desc. stats •Logistic regression •Pearson correlations	• 31% reported cancelling a health services appointment due to the COVID-19 outbreak • 30% of the participants cancelled an appointment to the oncology or haematology clinic because of the COVID-19 outbreak. Reasons were fear to contract the virus (93%), forgetfulness (4%), and lack of urgency (3%) • Contact with healthcare professionals was rated low on questionnaire • Perceived health status of half of the participants was moderate, and 35% of the participants had other additional diseases • The mean score of perceived susceptibility was moderate, while the mean score for anxiety was relatively low. Sense of mastery and social support were relatively high • Patients with perceived bad to reasonable health status, a lower sense of mastery, and higher anxiety had more contact with healthcare professionals during the COVID-19 pandemic • Participants who did not cancel an appointment to the oncology or haematology clinic during the COVID-19 outbreak perceived their health status as being bad to reasonable and had a higher sense of mastery and higher levels of social support • Participants who cancelled an appointment to the oncology or haematology clinic during the COVID-19 outbreak had higher perceived susceptibility and higher anxiety levels. Statistical decrease was found with being in contact with HC professional • About half of the participants reported being in isolation since the COVID-19 outbreak
Sigorski et al. (2020) Poland Multiple	1. To assess the relationship between the level of cancer-related anxiety (CRA) and SARS-CoV-2-related anxiety (SRA) among patients with cancer receiving anticancer systemic therapy 2. To distinguish subgroups of patients with the highest levels of anxiety and to assess strategy of coping with cancer	Patient Prospective, observational *N* = 306 > 18 years old	Primary data; survey Hospital setting 11 and 15 May 2020	•Questionnaire using: • Fear of COVID-19 Scale (SRA-FCV-19S) •Numerical Anxiety Scale (SRA-NAS) • Desc. stats	• Patients with breast cancer and treated with curative intention, as these factors are associated with a higher level of anxiety • The mean level of Fear of COVID was 18.5 (SD = 7.44), which was correlated with the Anxiety of COVID (*r* = 0.741, *p* < 0.001) • Fear of COVID was tumour type dependent • Anxiety observed in patients with breast cancer (17.63 ± 8.75) • Patients under 65 years old were associated with higher levels of anxiety • Anxiety related to cancer was higher in females
Singh et al. (2020) India Not specified	To assess the concerns and coping strategies and perspectives of patients suspected with COVID-19 at the National Cancer Institute	Patient Cross-sectional *N* = 103 Adults	Primary data: questionnaire Web-based April to May 2020	• Online questionnaire • Desc. stats	• 27% were COVID-19 asymptomatic • 33% of participants responded that they did need counselling • 55.3% were worried • 43% were anxious • 33% were sad • 46.6% were mostly comfortable • 14% were not stressed at all • 12% felt that their life had become difficult during the quarantine Coping mechanisms: • 80.6% reported support from family and friends • 71% remained connected to family and friends • 70% used spirituality/prayer • 45% used music therapy • 57% maintained a daily routine as a coping strategy • 2% were unable to cope
Souza et al. (2020) Brazil Breast	To understand the experience of cancer patients coping with COVID-19	Patient Qualitative participatory action *N* = 12 ≥ 18 years old	Primary data: virtual discussion Hospital setting June 2020	• Virtual culture circle • Thematic analysis	Two themes: 1. Challenges: cancer and COVID-19-Fear of infection, difficulty completing treatment, afraid to leave quarantine for treatment, anxious and concern for their health, stress, sadness 2. Learning: rising from one’s own ashes—Made them stronger, more united family, more time with family, faith, hope, opportunity to grow
Vanni et al. (2020) Italy Breast	To estimate the impact of anxiety among patients, caused by the COVID-19 pandemic	Patient Retrospective *N* = 160 39–80 years old	Primary data: interview Secondary data: medical notes Hospital setting 16th of January to the 20th of March 2020	• Medical notes • Interviewed via telephone • Literature review •Desc. stats • *T*-test • Fisher’s exact test	• Both POSTCOVID-19-Suspicious Breast Lesion and POST-COVID-19-Breast Cancer groups showed higher rates of procedure refusal and surgical refusal • Risk of COVID-19 Infection risk was the primary reason for refusal • Risk factors for surgical refusal include higher age at diagnosis, female gender, ethnicity, type of insurance, LABC (stage II and III BC), non-triple-negative breast cancer, residence areas with a low percentage of high school diplomas
Wang, Y. et al. (2020) China Not specified	To explore mental health problems in patients diagnosed with cancer during the COVID-19 pandemic	Patient Cross-sectional *N* = 6213 Adults	Primary data: interview Secondary data: electronic medical records Hospital setting 9 to 19 April 2020	• Interview using: • Visual Analogue Scale • WHOQOL-BRIEF scale • DSMIV-Insomnia Criteria •GAD-7 scale • PHQ-9 scale • Brief Symptom Inventory (BSI) • IES-R scale • Desc. stats • Linear regression	• 1.6% of patients were seeking help for psychological counselling • 48.1% did not pay attention to online mental health services • 11.2% considered online mental health services as helpful • Digestive system cancer and breast cancer showed a higher proportion of having mental health problems • 23.4% had depression • 17.7% had anxiety • 9.3% had PTSD •13.5% had hostility • Risk factors across different mental health problems, having a history of mental disorder, excessive alcohol consumption, having a higher frequency of worrying about cancer management due to COVID-19, feelings of overwhelming psychological pressure from COVID-19, high level of fatigue and pain • Inconvenience to go out for follow-up treatment was associated with higher risk of depression • Longer time since diagnosis, higher frequency of receiving COVID-19 information and news were associated with a higher level of PTSD symptoms • Females had a higher frequency of worrying about disease management due to COVID-19, increasing psychological pressure caused by COVID-19, and lower sleep quality • Younger age, male sex, being employed, longer time since diagnosis, receiving treatment, higher frequency of receiving COVID-19 information and news, satisfaction with personal health, good sleep quality, and having good relationships with friends were associated with lower risk of anxiety • Having been employed, longer time since diagnosis, and good sleep quality were associated with lower levels of depression • Younger age was a protective factor against hostility • Male sex, good sleep quality, and good relationships with friends were associated with lower levels of PTSD symptoms
Yan et al. (2020) USA Head and neck	1. Examine the impact of COVID-19 on head and neck cancer patients and advocacy organisations 2. Changes in patient concerns 3. Changes in HNC advocacy group programmes 3. Challenges faced	Patient and providers Qualitative *N* = 4 organisations for cancer patients NA	Primary data: interviews Organisation advocacy group Not specified	• Semi-structured interviews via phone and email • Thematic analysis	• Increased number of phone calls, emails, and messages on social media platforms contacting these organizations • Increased volume of calls involving COVID-19-related concerns Patient concerns: • Accessibility and/or delay of treatment • Risk of COVID-19 • Impact on cancer care • Inability to proceed with care alongside family members • Patients often may feel more isolated • Financial burden: worries about affording treatment and transportation to medical facilities
Yang, G. et al. (2020) China Multiple	To explore the effect of adverse childhood experience (ACE) on suicide ideation in young cancer patients during the COVID-19 pandemic	Patient Observational and cross-sectional *N* = 197 18–40 years old	Primary data; questionnaire Hospital setting January to May 2020	•Questionnaire using: • The self-rating Anxiety Scale (SAS) • The Pittsburgh Sleep Quality Index (PSQI) • The Beck Suicide Ideation Scale (BSI) • A blood biochemical examination to estimate inflammatory condition (CRP levels) •Desc. stats • Pearson correlation •Bootstrap analysis	• Young cancer patients demonstrated high levels of anxiety symptoms and suicide ideation, and low sleep quality, during the COVID-19 pandemic • Sleep quality, anxiety symptoms, and CRP levels affect suicidal ideation • ACE directly affected suicide ideation in young cancer patients • ACE affected suicide ideation directly and was mediated by roles sleep quality, anxiety symptom and CRP • ACE significantly and positively affected anxiety symptoms, CRP, and suicide ideation, but significantly affected sleep quality negatively • Anxiety symptoms significantly affected CRP levels and suicide ideation positively but significantly and negatively affected sleep quality • Sleep quality significantly and negatively affected suicide ideation, while CRP levels significantly and positively affected suicide ideation
Yang S. et al. (2020) China Lymphoma	1. To examine the impact of disrupted cancer care on anxiety and HRQoL of patients 2. Evaluate caregiver support and an online education programme of the Chinese Society of Clinical Oncology (CSCO)	Patient Cross-sectional *N* = 2532 subjects (1060 patients, 948 caregivers, and 524 members of the general public) > 20 years old	Primary data: questionnaire Web-based 17 to 19 April 2020	• Online questionnaire using: • Zung Self-Rating Anxiety Scale (SAS) • EORTC QLQ-C30 instrument •Desc. stats • *T*-test • ANOVA • *χ*^2^ tests • The Kendall tau-b correlation • Linear regression	• 56% of patients changed their routine hospital • 9% changed to a therapy of lower intensity • 4% switched to oral anti-lymphoma drugs • 13% delayed scheduled parenteral therapy • 37% delayed or postponed scheduled hospital visits • 24% experienced reduced therapy intensity including fewer drugs, reduced drug doses, a switch from parenteral to oral drugs, and/or therapy delay or discontinuation • 52% reported no change of their medical activities including physician visits, exams, and/or therapy • 33% if lymphoma patients had anxiety • Incidence of anxiety higher in lymphoma patients and their caregivers compared to members of the general public • More than 77% of respondents had minimal/moderate anxiety • Female sex, receiving therapy, reduced therapy intensity, and hospitalised patients were associated with more anxiety • Reduced therapy intensity was associated with worse HRQoL • Those who scored caregiver support and the online patient education programme high had better HRQoL • Paradoxically, lymphoma patients during the pandemic had better HRQoL than pre-pandemic controls • 39% were concerned about treatment disruption • 50% of patients were concerned about COVID-19 infection risk • Females were associated with more anxiety • Higher education level was associated with less anxiety • Social support resources for lymphoma patients included online patient support/discussion groups. Subjects who rated the quality of these online tools high had a better HRQoL
Yildirim et al. (2021) Turkey Multiple	1. To analyse anxiety and depression amongst cancer patients 2. To investigate the correlation between treatment delays and depression and anxiety levels in cancer patients	Patient Cross-sectional *N* = 637 first survey *N* = 595 s survey 18–76 years old	Primary data: questionnaire; secondary: medical notes Hospital setting Pre-pandemic survey from 3 to 22 February, 2020. Pandemic survey from 14 March to 5 July 2020	•Questionnaire • Medical records •Telephone interview/face to face using: • The Beck Depression Inventory (BDI) • The Beck Anxiety Inventory (BAI) •Desc. stats • Kolmogorov–Smirnov test • *T*-test • Mann–Whitney U test • ANOVA • Pearson’s correlation	• Depression and anxiety levels in cancer patients were found to increase during the pandemic • The increase was positively correlated with the disruption of their treatment (*p* = 0.000, *r* = 0.81) • Depression and anxiety levels and treatment delays were higher in elderly patients •Depression and anxiety levels were found to be significantly higher in females • Treatment delays were more common in patients who had to use public transportation • Elderly patients preferred to postpone their appointments for a while and stay home • Marital status, education level, social support, comorbidities, ECOG status, and stage of cancer were insignificant
Zuliani et al. (2020) Italy Not specified	To analyse how organisational changes related to SARSCoV-2 have impacted: (i) Volumes of oncological activity (compared to the same period in 2019), (ii) Hospital admissions of “active” oncological patients for SARS-CoV-2 infection	NA Retrospective *N* = 1 institution, *N* = 241 outpatients surveyed NA	Secondary data: health records Primary data: questionnaire Hospital setting 1 January to 31 March 2020 and 2019	• Medical records • Questionnaire on patient acceptance of measures • Desc. stats • *T*-test	Hospital admissions: • Jan–March 2020: reduced by 8% • Average weekly admissions showed a 40% reduction in March 2020 Chemotherapy admissions: • Jan–March 2020: reduced by 6% • 14% reduction in daily average Specialist visits: • Jan–March 2020: reduced by 3%. 35% reduction daily average visits, 7 patients were COVID positive • Almost all patients felt that the organisational measures adopted to minimise the risk of SARS-CoV-2 infection were clearly expressed (98%, 95%) • Acceptance of phone-based follow-ups and restaging visits, which were perceived as “not very adequate” (17%) or “not adequate at all” (18%) • Fear of accessing hospital facilities 34% • Fear that chemotherapy treatment could increase the risk of contracting SARS-CoV-2 infection 27%

## Results

**Fig. 1:**
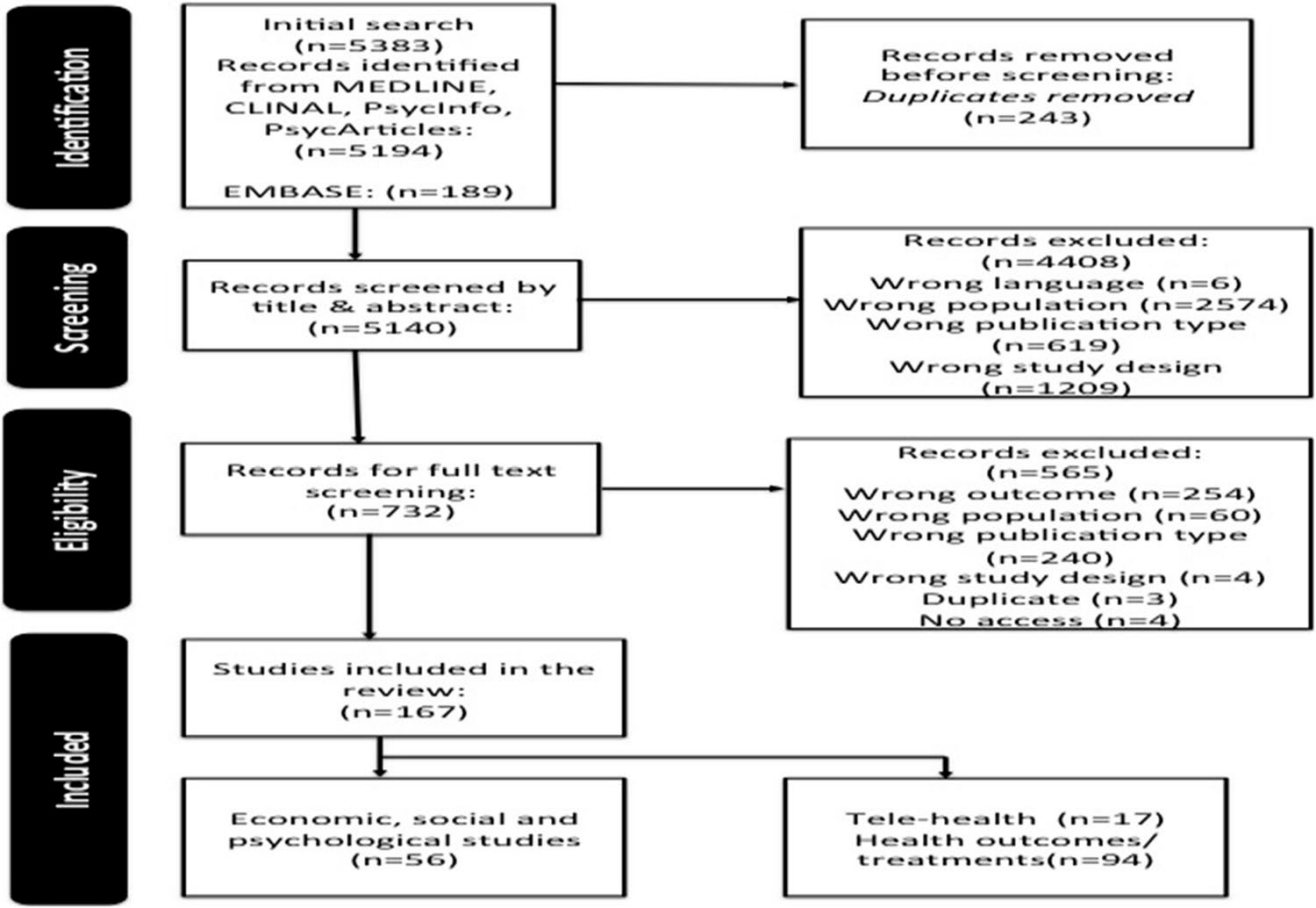
Flowchart of search output and results

In total 5383 references were imported into Rayyan, and 243 duplicates were removed. A total of 5140 records were screened by title and abstract by reviewers in two pairs (AL and AM; AK and FJD) and independently assessed against the inclusion criteria. In sum, 732 studies were identified for full-text review; 167 were considered for inclusion of which 56 report on the economic, social, and psychological impacts of COVID-19 [6] (see Fig. 1).

Of these, 96% were single-country studies, predominately from the USA (20%), Italy (18%), India (11%), and China (11%). The remainder were from elsewhere in Europe (19%), Middle East (11%), Asia (4%), Brazil (4%), or Canada (2%). Most studies included patients with multiple cancer types (64%). Others focus on specific cancer sites: breast (18%), gynaecological (5%), head and neck (3.6%), lung (3.6%), thyroid (1.8%), colorectal (1.8%), or lymphoma (1.8%).

### Social and psychological impacts of COVID-19 on cancer patients

#### Worry and fear

The most common mental health domain(s) identified were worry and/or fear around their cancer and getting COVID-19. Firstly, there was a heightened sense of fear of cancer recurrence or disease progression due to COVID-19-related disruptions or delays in cancer care [7–14]. Instruments used to measure fear and the level of fear experienced varied. For example, in a survey of 16 European countries, 71% of patients were concerned about cancer progression if their treatment/follow-up was cancelled/postponed [8]. In contrast, a survey in India reported fear of treatment delays and cancer progression as mostly moderate or minimal [10].

Secondly, there was fear and worry around getting COVID-19 amongst cancer patients [7–9, 11–13, 15–35]. A Singaporean study reported that 66% of patients had an elevated level fear of COVID-19 [36]. Gheorghe et al. [37] found that 68.5% of Romanian cancer patients were “very” or “somewhat worried” about COVID-19 compared to the influenza virus. Biagioli et al. [38] found that 37.3% of Italian cancer patients were “very or extremely” afraid of going to hospital because of an increased risk of contracting COVID-19 there, and 24.5% were “very or completely” afraid that their cancer care would become less important than being protected against COVID-19 infection, which would then have a negative impact on their prognosis. They also found that 53.8% believed they were at a higher risk of COVID-19 infection than the general population. Similarly, Erdem and Karaman [39] report that one-third of Turkish cancer patients were afraid to leave their house. These were more likely to be > 65 years, female, with stage 1 cancer, and with low education attainment. Those that did attend hospital appointments (~ 33%) were more likely to have stage 4 cancer; wore a mask (67%); and preferred not to use public transport owing to COVID-19 risk (95%). The majority (97%) did not accept visitors to their houses and washed their hands more often than usual (97.3%). Those finished treatment (radiotherapy or surgery) with co-morbidities (40%) were also afraid of COVID-19, in particular, females [7]. Higher levels of worry were found among females and older patients in Italy [16, 30], patients with comorbidities, immunocompromised and on active treatment in Denmark, the USA, and China [24, 28, 33], and amongst those with higher stress levels in Iran [40].

Patients tended to prioritise their cancer care over fear of contracting COVID-19, suggesting they were more afraid of delayed treatment and cancer progression than COVID-19 [10]. However, some sub-groups were willing to postpone/delay appointments and treatments [16, 21, 32].

Worryingly, as much as 61.8% were found to fear both COVID-19 infection and cancer progression equally [37]. In practice, the two fears/worries are intertwined, with patients reporting fear of getting COVID-19 infection when in hospital for treatment [11] and fearing that chemotherapy treatment could increase risk of COVID-19 infection (27% of Italian cancer patients sampled [41]). Disparity also existed amongst those who worried more about COVID-19 infection than their cancer, low socioeconomic groups [37], those undergoing palliative care [42], older [8, 16], frailer patients with co-morbidities [16], or those with a good understanding of immunosuppression [10].

#### Distress, anxiety, and depression

Košir et al. [9] conducted an international survey and found those currently or undergoing treatment within the last 6 months reporting higher levels of psychological distress on average; the most common concern being contracting COVID-19. Some American cancer patients found attending in-person appointments or treatment alone, and the cancellation or movement to virtual social support groups was emotionally challenging and distressing [26]. In France, being unemployed aggravated psychological distress further [43].

Several studies reported anxiety amongst samples of all cancer patients during the pandemic [29, 36, 42, 44]. Additionally, higher levels of anxiety were found amongst female patients in the UK, USA, Poland, and China [19, 26, 33, 35, 43, 45]; patients from lower income households [11]; older and more vulnerable patients in the Netherlands and Northern Italy [18, 30]; married patients in Singapore [36]; and patients with lower educational attainment in Italy, Singapore, and China [35, 36, 46]. In contrast, younger Chinese cancer patients demonstrated elevated levels of anxiety, suicide ideation, and low sleep quality, during the pandemic [47]. While in China, Wang et al. [33] found those employed had a lower risk of anxiety and depression.

Higher levels of depression were reported amongst female patients in China [43], older patients in Turkey [14], those from lower income households in South Korea and India [11, 44], patients who were unemployed in China [43], and unmarried non-parents in South Korea [11].

#### Social isolation and loneliness

Several studies reported social isolation and loneliness amongst cancer patients [9, 20, 38, 48, 49] which was mediated by detachment from loved ones, lack of social interaction, loneliness, fear of infection, worries about the future, and economic difficulties [38]. Several at-risk groups identified as feeling isolated and anxious. These included American women with ovarian cancer (10%) [20] and young cancer patients (aged 18–39) undergoing cancer care who felt more isolated (52%) than pre-pandemic, specifically missing social interactions [9]. Elran-Barak and Mozeikov [48] reported that lower levels of education and living without minor children in Israel were associated with feelings of loneliness and isolation. Leach et al. [26] in the USA found cancer patients experiencing social distancing and living alone had feelings of loneliness, while disruptions to cancer care were associated with increased loneliness and social isolation [26, 49]. Some patients’ perceived risk of COVID-19 infection caused them to engage in extreme levels of social isolation where they had no visitors and lived alone [26]. For others, the pandemic exasperated underlying situations. For example, Miaskowski et al. [49] upon comparing a more stressed group to a less stressed group found the former reported significantly higher levels of loneliness and social isolation and less resilience than the less stressed group.

#### Implications of social and psychological impacts

The review revealed that depression and anxiety progression might be associated with deliberate appointment delays [14]. When delays were at post-treatment follow-up or palliative care, this was exasperated further [42]. Also, Shinan‐Altman et al. [31] found that while incidences were low, patients with higher anxiety had more contact with healthcare professionals during the pandemic. Nevertheless, amongst the sample, anxiety did not directly influence healthcare decision-making.

Changes to the way healthcare organisations altered cancer care, to reduced COVID-19 transmission and increased uncertainty, for which tolerance and resilience varied. Hill et al. [23] found COVID-19 fear and intolerance of uncertainty were strongly correlated. Studies reported that health management changes to treatment plans increased patients’ psychological burden [9, 14, 20, 40, 43, 50–53]. These changes also increased uncertainty with an association between intolerance of uncertainty and anxiety, depressive symptoms, and stress [23]. While uncertainties can be reduced with additional information, health literacy is influenced by socio-economic factors. Erdem and Karaman [39] found that higher educational status was associated with better knowledge of COVID-19 transmission. Better knowledge of COVID-19 was associated with patients aged 40–54 years, in higher education, being female, living in urban areas, with a profession, and a higher income amongst a sample of Romanian cancer patients [37].

Collectively, adverse effects on specific mental health domains had a broad impact. For example, loneliness and isolation amongst cancer patients contributed to declines in general mental health [48] and translated into declines in QoL [15, 20]. Several studies found deterioration in QoL due to COVID-19 compounded by the effects of treatment and financial implications in terms of affordability of care [20, 26]. Despite some respondents being aware of and worried about their QoL [20], those with higher stress levels concerning COVID-19 had worse QoL [40]. Lower QoL and emotional functioning scores were correlated with concerns about contracting COVID-19 [24], with negative spill-over effects found on general health status and physical health in Italy [53] and Israel [48], respectively. Aggravating this further was postponement of care, which negatively impacted QoL [53]. Socio-economic differences were found here too. A higher QoL was correlated with patients who were older [54], employed, with fewer co-morbidities, and not receiving treatment within the last two months [24]. Patients with brain tumours, endometrial/cervical/vulva, and thoracic cancers had lower QoL scores [24]. Being female, having lower educational attainment, crowded housing conditions, and longer illness duration also negatively affected self-reported health status among patients in Israel [48].

### Economic impact of COVID-19 on cancer patients and care

#### Out-of-pocket expenses

From an individual perspective where treatments are not covered under government schemes or insurance, patients experience a large financial burden, in-terms of out-of-pocket expenses, expensive health insurance premiums, or deductibles [26]. This was the case for a considerable proportion of cancer patients in India [44] and Jordan [50]. While this is not a new phenomenon, it has been exasperated by COVID-19. Modified workflows and redistributed cancer care resources saw widespread use of telemedicine, with mixed effects. Transition to telemedicine saved US patients’ time and money ($170) [55]. In contrast, Bakkar et al. [50] found that treatment changes incurred additional patient costs in Jordan. Globally, testing for COVID-19 (negative results were required to attend appointments) introduced varying fee structures with additional costs experienced amongst cancer patients in India [56] and South Korea [11]. These costs affected patients’ decision to undergo cancer treatment particularly in poorer countries [56].

#### Household finances

Widespread lockdowns during COVID-19 Wave I caused an economic downturn, with negative implications on labour markets. Cancer patients in India (22.2%) expressed fears around losing their jobs and the implications of the expected economic crisis for their family [13]. Similarly, in the USA, cancer patients reported concerns with maintaining employment. These economic conditions exasperated pre-existing cancer financial burdens, including paying for prescriptions and high insurance deductibles in private healthcare markets in the USA [26]. US patients were also concerned with affordability of treatments and transportation to medical facilities [34]. Reduced earnings caused some patients to choose not to attend medical appointments. For example, 46% of cancer patients in India reported financial difficulties as the primary reason for missed consultations [57].

Several studies identified socio-economic factors associated with the economic impacts of COVID-19. Patients that were unemployed, with low educational attainment, or with lower incomes, or were under financial pressure had greater difficulties accessing care in India [44]. In the USA, non-Hispanic white, married, more educated, and older cancer patients were less likely to cite financial worries [26]. These socio-economic inequalities coupled with increased financial burden create barriers to accessing care [44].

#### Payers and providers

The consequences of COVID-19-related healthcare changes are potentially worsening patients’ clinical condition, which also has economic consequences at the provider level with implications for patients. For example, there is an increased likelihood of post-operative ICU following delayed surgery [58]. During the pandemic, demands for services like respiratory assistance increased. This dual increase in demand had resource implications for healthcare providers; reduced resources limit choices to delivering post-operative cancer care or care for COVID-19 patients [59].

The introduction and expansion of telemedicine during the pandemic had mixed effects for healthcare providers and payers. Parikh et al. [55] reported the transition to telemedicine saved hospitals time and money in the USA. However, Goenka et al. [60] report that while it did ensure continuation of care, its cost and financial impacts must be considered. It may not be financially suitable for all care due to implementation and maintenance costs compared with reimbursement received, cost savings to patients (direct and indirect), and care coordination. Mahl et al. [42] found similar results for tele-consultation technologies for palliative and follow-up services in Brazil. Furthermore, the high prevalence of anxieties and fears discussed above culminated in increased demand for support services and information, as demonstrated by the increased volume of calls, emails, and messages found in one North American study [34]. One Italian study found such communications tended to have more negative than positive messages (57% vs 43%) [21].

### Innovations to mitigate impacts

Social and psychological impacts, along with modified workflows that redistributed healthcare resources, had an economic impact on patients and healthcare systems. US patients experiencing higher stress had more financial concerns [49], and COVID-19 exasperated existing financial burden associated with cancer on families, with some not being able to afford to attend consultations [57]. Studies revealed coping strategies adopted by cancer survivors and patients to deal with the impact of the pandemic [20, 61]. Mechanisms used to cope included connection to family and friends, spirituality/prayer, music therapy, emotional supports, positive reframing, and daily routine, including self-care, hobbies, and planning [30, 61, 62]. These strategies were an important predictor of QoL [20] and positive health behaviours. Positive lifestyle choices were likely to alleviate mental health burden and improve HRQoL [30, 35, 54], while others employed avoidance coping strategies, including self-distraction and substance use [62].

Using positive coping strategies was important as delivery of support services was adversely impacted by COVID-19 [13, 51, 61]. Such supports included psychological and peer group support services, counseling for patients [13, 51, 61], and caregiver supports [35]. Maintaining or quickly resuming these supports during the pandemic was associated with less anxiety [35] and distress [51]. Additionally, patients with higher social support had more contact with healthcare professionals during the COVID-19 pandemic [31].

## Discussion

Economic burden associated with cancer for patients is measured through direct and indirect costs with both objective (i.e. financial burden) and subjective approaches (financial distress) [3]. This adjustment to economic burden measurement coincides with the growing knowledge of cancer as a chronic disease, which widens perspective and shifts understanding of possible side effects beyond clinical outcomes.

Pre-pandemic psychological strain was caused by possible cancer treatment side effects which have adverse health outcomes [63]. The pandemic exasperated these health outcomes further. National and institutional public health guidelines to reduce COVID-19 transmission resulted in suspended cancer screening programmes, delayed diagnoses, postponed or deferred treatments, and altered treatment regimes in many countries. These health system shocks also altered patients’ decision making and health-seeking behaviours. For example, to shield from COVID-19, patients delayed seeking medical appointments, avoided clinical settings, etc. In the immediate and long term, this has direct impacts on health outcomes, increasing cancer morbidity and mortality. Longer-term strategic outcomes are also hampered with deferred cancer strategies that have direct consequences for achieving high-level targets such as the United Nations' (UN) Sustainable Development Goal 3 of good health and well-being for all and the European Commission Cancer Mission of improving the lives of those affected by cancer through prevention and cure.

Collectively, these types of costs can affect patients’ QoL that represents an additional cost to their well-being [3, 64, 65]. This review predominately captures the experience during the first wave of the pandemic, when national lockdowns and public health guidelines were at the highest level internationally. So while many of the impacts and costs from previous frameworks [3] were identified in the review, the studies adopted a short-term perspective. We suspect that as the pandemic persists, and more longitudinal data becomes available, further costs and impacts will be documented capturing a broader range of costs, for example, re-distribution of resources in healthcare delivery and subsequent opportunity costs of telemedicine from a patient and provider perspective, the associated treatment costs of COVID-19, or the feelings of isolation that affected QoL.

Despite gaps in range and severity of costs, evidence from this review demonstrates the demand for psychological support and information to alleviate fears and provide reassurance to patients. COVID-19 increased information asymmetries in the delivery of all healthcare services, including cancer care. Imperfect and inadequate information create uncertainties which influence patients’ behaviour and decision-making. With poor information and high levels of uncertainty, patients were risk adverse, reducing their health-seeking behaviours, avoiding public transport, clinical settings, etc. Such behaviours have long-term impacts; later diagnosis and delayed treatment adversely impact cancer patients’ health outcomes, QoL, and mortality.

While economies and healthcare systems are recovering, COVID-19 remains a threat for cancer patients. Accurate and effective information and support for cancer patients are still needed to mitigate further impacts and to support patients in its aftermath. In some settings, tele-health could be used to provide this; however, a one-size-fits-all approach is not suitable. Supports and information need to be tailored to the target audience, depending on the healthcare system characteristics, resources available, and the socio-economic profile of the patient population. This is imperative as we emerge from the pandemic, to mitigate its affects, promote recovery in health systems and in patients, and implement learnings for future possible pandemics.

The review revealed several approaches to minimise the economic, social, and psychological impacts of COVID-19 for cancer patients, centring around provision of information and support services and coping strategies. Regarding information, three recommendations emerged. Firstly, more information is required, specifically with regard to how to cope with the pandemic [9]. Secondly, effective communication between physicians and patients is required [14]. Effectively delivered communications provide valuable information that can reduce information asymmetries. Thirdly, information could be provided via online education programmes, which may alleviate anxiety and improve HRQoL [35], as appropriately designed interventions can reduce cancer-related psychosocial outcomes and quality of life [66, 67].

### Limitations

This review mainly captured the first wave of the COVID-19 pandemic in March 2020. Many studies were reliant on single institutions (e.g. [44, 55]) and lack long-term follow-up [55]. In some cases, data were only available for short periods (e.g. [44, 59]) which had negative implications on sample size. Data collection methods varied, with many using online methods that can exclude those experiencing technology barriers.

## Conclusion

COVID-19 compounded the economic, social, and psychological impacts of cancer on patients owing to health system adjustments and reduction in economic activity. Identification of the impact of COVID-19 on cancer patients from a psychological, social, and economic perspective following the pandemic can inform the design of timely and appropriate interventions and supports, to deal with the backlog in cancer care and enhance recovery. While the long-term effects and economic fallout for cancer patients are not easily quantified, they are real and further support is required to alleviate the issues cancer patients face.

## Supplementary Information

Below is the link to the electronic supplementary material.

Supplementary file1 (DOCX 20 kb)

Supplementary file2 (DOCX 41 kb)

## Data Availability

Materials and codes available upon request to the authors.

## References

[1] Sung H, Ferlay J, Siegel R, Laversanne M, Soerjomataram I, Jemal A, et al. 2020 Global cancer statistics. GLOBOCAN estimates of incidence and mortality worldwide for. 36. 10.3322/caac.21660 33538338

[2] Stewart BW, Wild CP(ed). World Cancer Report Lyon: WHO press; 2014 [Available from: https://publications.iarc.fr/Non-Series-Publications/World-Cancer-Reports/World-Cancer-Report-2014.

[3] Essue BM, Iragorri N, Fitzgerald N, de Oliveira C (2020). The psychosocial cost burden of cancer: a systematic literature review. Psychooncology.

[4] Higgins JP. Cochrane handbook for systematic reviews of interventions version 5.0. 1. The Cochrane Collaboration. http://www.cochrane-handbook org. 2008.

[5] Davies KS (2011). Formulating the evidence based practice question: a review of the frameworks. Evid Based Libr Inf Pract.

[6] Murphy A, Lawlor, A., Kirby, A. Drummond, F.D. 2021 A systematic review of the impact of COVID-19 pandemic on cancer patients and survivors from an economic and social perspective. University College Cork, Cork, Ireland.

[7] Catania C, Spitaleri G, Del Signore E, Attili I, Radice D, Stati V, et al. 2020 Fears and perception of the impact of COVID-19 on patients with lung cancer: a mono-institutional survey. Frontiers Oncol 10. 10.3389/fonc.2020.584612 PMC7591454 33163413

[8] Gultekin M, Ak S, Ayhan A, Strojna A, Pletnev A, Fagotti A (2021). Perspectives, fears and expectations of patients with gynaecological cancers during the COVID-19 pandemic: a Pan-European study of the European Network of Gynaecological Cancer Advocacy Groups (ENGAGe). Cancer Med.

[9] Košir U, Loades M, Wild J, Wiedemann M, Krajnc A, Roškar S (2020). The impact of COVID-19 on the cancer care of adolescents and young adults and their well-being: results from an online survey conducted in the early stages of the pandemic. Cancer.

[10] Ghosh J, Ganguly S, Mondal D, Pandey P, Dabkara D, Biswas B (2020). Perspective of oncology patients during COVID-19 pandemic: a prospective observational study from India. JCO Global Oncol.

[11] Kim SY, Kim S. 2021. Do COVID-19-related treatment changes influence fear of cancer recurrence, anxiety, and depression in breast cancer patients? Cancer nursing. 10.1097/NCC.0000000000000937 33654008

[12] Lou E, Teoh D, Brown K, Blaes A, Holtan SG, Jewett P (2020). Perspectives of cancer patients and their health during the COVID-19 pandemic. PLoS ONE.

[13] Mitra M, Basu M (2020). A study on challenges to health care delivery faced by cancer patients in India during the COVID-19 pandemic. J Prim Care Commun Health.

[14] Yildirim OA, Poyraz K, Erdur E. 2021 Depression and anxiety in cancer patients before and during the SARS-CoV-2 pandemic: association with treatment delays. Qual Life Res: Int J Qual Life Asp Treat Care Rehabil 10.1007/s11136-021-02795-4 PMC7907665 33635508

[15] Bäuerle A, Musche V, Schmidt K, Schweda A, Fink M, Weismüller B (2021). Mental health burden of german cancer patients before and after the outbreak of COVID-19: predictors of mental health impairment. Int J Environ Res Public Health.

[16] Campi R, Tellini R, Grosso AA, Amparore D, Mari A, Viola L (2021). Deferring elective urologic surgery during the COVID-19 pandemic: the patients' perspective. Urology.

[17] Chia JMX, Goh ZZS, Chua ZY, Ng KYY, Ishak D, Fung SM (2021). Managing cancer in context of pandemic: a qualitative study to explore the emotional and behavioural responses of patients with cancer and their caregivers to COVID-19. BMJ Open.

[18] de Joode K, Dumoulin DW, Engelen V, Bloemendal HJ, Verheij M, van Laarhoven HWM (2020). Impact of the coronavirus disease 2019 pandemic on cancer treatment: the patients' perspective. Eur J Cancer.

[19] Fox L, Wylie H, Cahill F, Haire A, Green S, Kibaru J (2021). Gender differences in concerns about participating in cancer research during the COVID-19 pandemic. Cancer control J Moffitt Cancer Center.

[20] Frey MK, Ellis AE, Zeligs K, Chapman-Davis E, Thomas C, Christos PJ (2020). Impact of the coronavirus disease 2019 pandemic on the quality of life for women with ovarian cancer. Am J Obstet Gynecol.

[21] Gebbia V, Piazza D, Valerio MR, Borsellino N, Firenze A (2020). Patients with cancer and COVID-19: a WhatsApp messenger-based survey of patients' queries, needs, fears, and actions taken. JCO global oncology.

[22] Han J, Zhou F, Zhang L, Su Y, Mao L (2021). Psychological symptoms of cancer survivors during the COVID-19 outbreak: a longitudinal study. Psychooncology.

[23] Hill EM, Frost A, Martin JD. 2021 Experiences of women with ovarian cancer during the covid-19 pandemic: examining intolerance of uncertainty and fear of covid-19 in relation to psychological distress. J Psychosoc Oncol 10.1080/07347332.2021.1880524 33559539

[24] Jeppesen SS, Bentsen KK, Jørgensen TL, Holm HS, Holst-Christensen L, Tarpgaard LS (2021). Quality of life in patients with cancer during the COVID-19 pandemic â€“ a Danish cross-sectional study (COPICADS). Acta Oncol.

[25] Kamposioras K, Saunders M, Jonathan Lim KH, Marti K, Anderson D, Cutting M, 2020 et al. The impact of changes in service delivery in patients with colorectal cancer during the initial phase of the COVID-19 pandemic. Clin Colorectal Cancer 10.1016/j.clcc.2020.11.006 PMC7718777 33384244

[26] Leach CR, Kirkland EG, Masters M, Sloan K, Rees-Punia E, Patel AV, et al. 2021 Cancer survivor worries about treatment disruption and detrimental health outcomes due to the COVID-19 pandemic. J Psychosoc Oncol 10.1080/07347332.2021.1888184 33624572

[27] Ng KYYZS, Tan SH, Ishak NDB, Goh ZZS, Chua ZY, Chia JMX, Chew EL, Shwe T, Mok JKY, Leong SS, Lo JSY, Ang ZLT, Leow JL, Lam CWJ, Kwek JW, Dent R, Tuan J, Lim ST, Hwang WYK, Griva K, Ngeow J (2020). Understanding the psychological impact of COVID-19 pandemic on patients with cancer, their caregivers, and health care workers in Singapore. JCO Global Oncol.

[28] Papautsky EL, Hamlish T (2021). Emotional response of US breast cancer survivors during the COVID-19 pandemic. Cancer Invest.

[29] Philip KEJ, Lonergan B, Cumella A, Farrington-Douglas J, Laffan M, Hopkinson NS (2020). COVID-19 related concerns of people with long-term respiratory conditions: a qualitative study. BMC Pulm Med.

[30] Pigozzi E, Tregnago D, Costa L, Insolda J, Turati E, Rimondini M (2021). Psychological impact of Covid-19 pandemic on oncological patients: a survey in Northern Italy. PLoS ONE.

[31] Shinan-Altman S, Levkovich I, Tavori G (2020). Healthcare utilization among breast cancer patients during the COVID-19 outbreak. Palliat Support Care.

[32] Vanni G, Materazzo M, Pellicciaro M, Ingallinella S, Rho M, Santori F (2020). Breast cancer and COVID-19: the effect of fear on patients' decision-making process. In vivo (Athens, Greece).

[33] Wang Y, Duan Z, Ma Z, Mao Y, Li X, Wilson A (2020). Epidemiology of mental health problems among patients with cancer during COVID-19 pandemic. Transl Psychiatry.

[34] Yan F, Rauscher E, Hollinger A, Caputo MA, Ready J, Fakhry C (2020). The role of head and neck cancer advocacy organizations during the COVID-19 pandemic. Head Neck.

[35] Yang S, Dong D, Gu H, Gale RP, Ma J, Huang X. 2020 Impact of stopping therapy during the SARS-CoV-2 pandemic in persons with lymphoma. J Cancer Res Clin Oncol 10.1007/s00432-020-03426-0 PMC7571863 33078214

[36] Ng KYY, Zhou S, Tan SH, Ishak NDB, Goh ZZS, Chua ZY (2020). Understanding the psychological impact of COVID-19 pandemic on patients with cancer, their caregivers, and health care workers in Singapore. JCO Global Oncol.

[37] Gheorghe AS, Negru ŞM, Niţipir C, Mazilu L, Marinca M, Gafton B (2021). Knowledge, attitudes and practices related to the COVID-19 outbreak among Romanian adults with cancer: a cross-sectional national survey. ESMO Open.

[38] Biagioli V, Albanesi B, Belloni S, Piredda A, Caruso R (2021). Living with cancer in the COVIDâ 19 pandemic: an Italian survey on selfâ isolation at home. Eur J Cancer Care.

[39] Erdem D, Karaman I (2020). Awareness and perceptions related to COVIDâ 19 among cancer patients: a survey in oncology department. Eur J Cancer Care.

[40] Charsouei S, Esfahlani MZ, Dorosti A, Zamiri RE (2021). Effects of COVID-19 pandemic on perceived stress, quality of life, and coping strategies of women with breast cancer with spinal metastasis under chemotherapy. Int J Women's Health Reprod Sci.

[41] Zuliani S, Zampiva I, Tregnago D, Casali M, Cavaliere A, Fumagalli A (2020). Organisational challenges, volumes of oncological activity and patients' perception during the severe acute respiratory syndrome coronavirus 2 epidemic. Eur J Cancer.

[42] Mahl C, Melo LRS, Almeida MHA, Carvalho CS, Santos LLS, Nunes PS (2020). Delay in head and neck cancer care during the COVID-19 pandemic and its impact on health outcomes. Braz Oral Res.

[43] Chaix B, Delamon G, Guillemassé A, Brouard B, Bibault J-E (2020). Psychological distress during the COVID-19 pandemic in France: a national assessment of at-risk populations. Gen Psychiatr.

[44] Rajan S, Akhtar N, Tripathi A, Kumar V, Chaturvedi A, Mishra P (2021). Impact of COVID-19 pandemic on cancer surgery: patient's perspective. J Surg Oncol.

[45] Sigorski D, Sobczuk P, Osmola M, Kuć K, Walerzak A, Wilk M (2020). Impact of COVID-19 on anxiety levels among patients with cancer actively treated with systemic therapy. ESMO Open..

[46] Merz V, Ferro A, Piras EM, Zanutto A, Caffo O, Messina C (2021). Electronic medical record-assisted telephone follow-up of breast cancer survivors during the COVID-19 pandemic: a single institution experience. JCO Oncol Pract.

[47] Yang G, Xiao C, Li S, Yang N (2020). The effect and mechanism of adverse childhood experience on suicide ideation in young cancer patients during coronavirus disease 2019 (COVID-19) pandemic. Risk Manag Healthc Policy.

[48] Elran-Barak R, Mozeikov M (2020). One month into the reinforcement of social distancing due to the COVID-19 outbreak: subjective health, health behaviors, and loneliness among people with chronic medical conditions. Int J Environ Res Public Health.

[49] Miaskowski C, Paul SM, Snowberg K, Abbott M, Borno H, Chang S (2020). Stress and symptom burden in oncology patients during the COVID-19 pandemic. J Pain Symptom Manage.

[50] Bakkar S, Al-Omar K, Aljarrah Q, Al-Dabbas Md, Al-Dabbas N, Samara S (2020). Impact of COVID-19 on thyroid cancer surgery and adjunct therapy. Updat Surg.

[51] Juanjuan L, Santa-Maria CA, Hongfang F, Lingcheng W, Pengcheng Z, Yuanbing X (2020). Patient-reported outcomes of patients with breast cancer during the COVID-19 outbreak in the epicenter of China: a cross-sectional survey study. Clin Breast Cancer.

[52] Massicotte V, Hans I, Savard J (2021). COVID-19 pandemic stressors and psychological symptoms in breast cancer patients. Curr Oncol (Toronto, Ont).

[53] Greco F, Altieri VM, Esperto F, Mirone V, Scarpa RM. 2020 Impact of COVID-19 pandemic on health-related quality of life in uro-oncologic patients: what should we wait for? Clinical Genitourinary Cancer. 10.1016/j.clgc.2020.07.008 PMC7366083 32863188

[54] Baffert K-A, Darbas T, Lebrun-Ly V, Pestre-Munier J, Peyramaure C, Descours C (2021). Quality of life of patients with cancer during the COVID-19 pandemic. In vivo (Athens, Greece).

[55] Parikh NR, Chang EM, Kishan AU, Kaprealian TB, Steinberg ML, Raldow AC (2020). Time-driven activity-based costing analysis of telemedicine services in radiation oncology. Int J Radiat Oncol Biol Phys.

[56] Deshmukh S, Naik S, Zade B, Patwa R, Gawande J, Watgaonkar R (2020). Impact of the pandemic on cancer care: lessons learnt from a rural cancer center in the first 3 months. J Surg Oncol.

[57] Akhtar N, Rajan S, Chakrabarti D, Kumar V, Gupta S, Misra S (2021). Continuing cancer surgery through the first six months of the COVID-19 pandemic at an academic university hospital in India: a lower-middle-income country experience. J Surg Oncol.

[58] Sud A, Jones ME, Broggio J, Loveday C, Torr B, Garrett A (2020). Collateral damage: the impact on outcomes from cancer surgery of the COVID-19 pandemic. Ann Oncol Off J Eur Soc Med Oncol.

[59] Mari G, Giordano R, Uccelli M, Cesana G, Olmi S, Ferrari GC (2020). Do we really know how much the COVID-19 pandemic affected the surgical practice in Northern Italy? A multi-center comparative study and cost analysis. Chirurgia (Bucharest, Romania : 1990).

[60] Goenka A, Ma D, Teckie S, Alfano C, Bloom B, Hwang J (2021). Implementation of telehealth in radiation oncology: rapid integration during COVID-19 and its future role in our practice. Adv Radiat Oncol.

[61] Singh N, Kumar S, Rathore P, Vig S, Vallath N, Mohan A (2020). Concerns and coping strategies of persons under institutional quarantine during SARS-CoV-2 pandemic. Indian J Palliat Care.

[62] Frey MK, Chapman-Davis E, Glynn SM, Lin J, Ellis AE, Tomita S (2021). Adapting and avoiding coping strategies for women with ovarian cancer during the COVID-19 pandemic. Gynecol Oncol.

[63] Carrera PM, Kantarjian HM, Blinder VS (2018). The financial burden and distress of patients with cancer: understanding and stepping-up action on the financial toxicity of cancer treatment. CA Cancer J Clin.

[64] Lathan CS, Cronin A, Tucker-Seeley R, Zafar SY, Ayanian JZ, Schrag D (2016). Association of financial strain with symptom burden and quality of life for patients with lung or colorectal cancer. J Clin Oncol.

[65] Tompa E, Kalcevich C, McLeod C, Lebeau M, Song C, McLeod K (2017). The economic burden of lung cancer and mesothelioma due to occupational and para-occupational asbestos exposure. Occup Environ Med.

[66] Nikita, Rani R, Kumar R. 2022 Body image distress among cancer patients: needs for psychosocial intervention development. Supportive Care in Cancer 10.1007/s00520-022-07049-8 PMC9002224 35412075

[67] Rawl SM, Given BA, Given CW, Champion VL, Kozachik SL, Barton D, et al., editors. 2002. Intervention to improve psychological functioning for newly diagnosed patients with cancer. Oncology Nursing Forum 10.1188/02.ONF.967-975 12096294

