# Supplementary material for: Counting the social, psychological, and economic costs of COVID-19 for cancer patients
Source: Support Care Cancer. 2022 Jun 11;30(11):8705–31. doi: 10.1007/s00520-022-07178-0 (PMC9188420; doi:10.1007/s00520-022-07178-0)
Supplement: Supplementary file 1 — Supplementary file1 (DOCX 20 kb) [file 520_2022_7178_MOESM1_ESM.docx]

Appendix 1

EBSCO search terms

1. "covid-19"
2. "coronavirus"
3. "2019-ncov"
4. "sars-cov-2"
5. "cov-19"
6. "severe acute respiratory syndrome coronavirus-2"
7. "pandemic"
8. #1 OR #2 OR #3 OR #4 OR #5 OR #6 OR #7
9. MH "SARS-CoV-2"
10. MH "COVID-19"
11. MH "Pandemics"
12. #9 OR #10 OR #11
13. #8 OR #12
14. MH "Neoplasms
15. "cancer"
16. "oncology"
17. "malignant"
18. "tumour"
19. "metastasis"
20. "neoplasm"
21. #15 OR #16 OR #17 OR #18 OR #19 OR #20
22. #14 OR #21
23. "quality of life"
24. "health-related quality of life"
25. "survival"
26. "mortality"
27. "disease progression"
28. "diagnosis"
29. "screening"
30. "recurrence"
31. "disease stage"
32. "delay"
33. "support"
34. "surgery"
35. "treatment"
36. "target therapy"
37. "radiotherapy"
38. "chemotherapy"
39. "immunotherapy"
40. "hormone therapy"
41. "survivorship programme"
42. "follow-up-care"
43. "financial toxicity"
44. "out-of-pocket"
45. "productivity"
46. "absenteeism"
47. "unemployment"
48. "cost"
49. "waiting time"
50. "expenses"
51. "financial stress"
52. "inconvenience"
53. "opportunity cost"
54. "income"
55. "well being"
56. "social isolation"
57. "exclusion"
58. "loneliness"
59. "happiness"
60. "life satisfaction"
61. "fatigue"
62. "insomnia"
63. "psychological distress"
64. "emotional distress"
65. "anxiety"
66. "depression"
67. "post-traumatic stress disorder"
68. "cancer care"
69. "financial"
70. "survivors"
71. "psychological"
72. #23 OR #24 OR #25 OR #26 OR #27 OR #28 OR #29 OR #30 OR #31 OR #32 OR #33 OR #34 OR #35 OR #36 OR #37 OR #38 OR #39 OR #40 OR #41 OR #42 OR #43 OR #44 OR #45 OR #46 OR #47 OR #48 OR #49 OR #50 #51 OR #52 OR #53 OR #54 OR #55 OR #56 OR #57 OR #58 OR #59 OR #60 OR #61 OR #62 OR #63 OR #64 OR #65 OR #66 OR #67 OR #68 OR #69 OR #70 OR #71
73. MH "Neoplasms/MO/DI/DT/EC/TH/RT/PX/CL/SU"
74. MH "Neoplasm Staging"
75. MH "Neoplasm Recurrence, Local"
76. MH "Delayed Diagnosis"
77. MH "Cost of Illness"
78. MH "Cancer Survivors"
79. MH "Anxiety"
80. MH "Depression"
81. MH "Early Detection of Cancer"
82. MH "Delivery of Health Care"
83. MH "Quality of Life"
84. MH "Survivorship"
85. MH "Stress, Psychological"
86. MH "Unemployment"
87. MH "Social Isolation"
88. MH "Psychological Distress"
89. MH "Loneliness"
90. MH "Happiness"
91. MH "Economic"
92. MH "Socioeconomic Factors"
93. MH "Disease progression"
94. MH “Fatigue”
95. MH “Insomnia”
96. MH “After Care”
97. MM “Mortality”
98. MH "Health Expenditures"
99. MH "Absenteeism"
100. MH "Efficiency"
101. MH "Costs and Cost Analysis"
102. MH "Income"
103. MH "Social Change"
104. MH "Economics"
105. MH "Financial Support"
106. MH "Financial Stress"
107. MH "Social Support"
108. MH "Psychosocial Support Systems"
109. MH "Stress Disorders, Post-Traumatic"
110. MH "Value of Life"
111. MH "Personal Satisfaction"
112. #73 OR #74 OR #75 OR #76 OR #77 OR #78 OR #79 OR #80 OR #81 OR #82 OR #83 OR #84 OR #85 OR #86 OR #87 OR #88 OR #89 OR #90 OR #91 OR #92 OR #93 OR #94 OR #95 OR #96 OR #97 OR #98 OR #99 OR #100 OR #101 OR #102 OR #103 OR #104 OR #105 OR #106 OR #107 OR #108 OR #109 OR #110 OR #111
113. #72 OR #112
114. #13 AND #22 AND #113

Appendix 2

EMBASE search terms

1. ‘coronavirus disease 2019’:ab,ti,kw
2. ‘severe acute respiratory syndrome 2’:ab,ti,kw,
3. #1 OR #2
4. ‘malignant neoplasm’:ab,ti,kw
5. ‘neoplasm’:ab,ti,kw
6. ‘metastasis’:ab,ti,kw
7. ‘oncology’:ab,ti,kw
8. #4 OR #5 OR #6 OR #7
9. ‘quality of life’:ab,ti,kw
10. ‘survival’:ab,ti,kw
11. ‘mortality’:ab,ti,kw
12. ‘disease exacerbation’:ab,ti,kw
13. ‘diagnosis’:ab,ti,kw
14. ‘cancer recurrence’:ab,ti,kw
15. ‘cancer staging’:ab,ti,kw
16. ‘social support’:ab,ti,kw
17. ‘surgery’:ab,ti,kw
18. ‘therapy’:ab,ti,kw
19. ‘chemotherapy’:ab,ti,kw
20. ‘radiotherapy’:ab,ti,kw
21. ‘cancer therapy’:ab,ti,kw
22. ‘hormonal therapy’:ab,ti,kw
23. ‘immunotherapy’:ab,ti,kw
24. ‘survivorship’:ab,ti,kw
25. ‘cancer survivor’:ab,ti,kw
26. ‘financial stress’:ab,ti,kw
27. ‘out of pocket costs’:ab,ti,kw
28. ‘absenteeism’:ab,ti,kw
29. ‘productivity’:ab,ti,kw
30. ‘unemployment’:ab,ti,kw
31. ‘cost’:ab,ti,kw
32. ‘income’:ab,ti,kw
33. ‘waiting time’:ab,ti,kw
34. ‘loneliness’:ab,ti,kw
35. ‘social exclusion’:ab,ti,kw
36. ‘happiness’:ab,ti,kw
37. ‘satisfaction’:ab,ti,kw
38. ‘fatigue’:ab,ti,kw
39. ‘insomnia’:ab,ti,kw
40. ‘distress symdrome’:ab,ti,kw
41. ‘emotional stress’:ab,ti,kw
42. ‘anxiety’:ab,ti,kw
43. ‘depression’:ab,ti,kw
44. ‘posttraumatic stress disorder’:ab,ti,kw
45. ‘delayed diagnosis’:ab,ti,kw
46. ‘aftercare’:ab,ti,kw
47. #9 OR #10 OR #11 OR #12 OR #13 OR #14 OR #15 OR #16 OR #17 OR #18 OR #19 OR #20 OR #21 OR #22 OR #23 OR #24 OR #25 OR #26 OR #27 OR #28 OR #29 OR #30 OR #31 OR #32 OR #33 OR #34 OR #35 OR #36 OR #37 OR #38 OR #39 OR #40 OR #41 OR #42 OR #43 OR #44 OR #45 OR #46
48. #3 AND #8 AND #47
