# Supplementary material for: Counting the social, psychological, and economic costs of COVID-19 for cancer patients
Source: Support Care Cancer. 2022 Jun 11;30(11):8705–31. doi: 10.1007/s00520-022-07178-0 (PMC9188420; doi:10.1007/s00520-022-07178-0)
Supplement: Supplementary file 2 — Supplementary file2 (DOCX 41 kb) [file 520_2022_7178_MOESM2_ESM.docx]

**APPENDIX 3 Quality Review**

| **I PREVALENCE STUDIES**  1. Was the sample frame appropriate to address the target population?  2. Were study participants sampled in an appropriate way?  3. Was the sample size adequate?  4.Were the study subjects and the setting described in detail?  5.Was the data analysis conducted with sufficient coverage of the identified sample?  6. Were valid methods used for the identification of the condition?  7. Was the condition measured in a standard, reliable way for all participants?  8. Was there appropriate statistical analysis?  9. Was the response rate adequate, and if not, was the low response rate managed appropriately? | | | | | | | | | |
| --- | --- | --- | --- | --- | --- | --- | --- | --- | --- |
| **Author** | **Q1** | **Q2** | **Q3** | **Q4** | **Q5** | **Q6** | **Q7** | **Q8** | **Q9** |
| Baffert et al. (2021) | Y | UC | UC | Y | Y | Y | Y | Y | UC |
| Bakkar et al. (2020) | Y | UC | UC | Y | UC | Y | Y | Y | UC |
| Bauerle et al. (2020) | Y | UC | UC | Y | Y | Y | Y | Y | UC |
| Biagioli et al. (2020) | UC | UC | UC | Y | Y | Y | Y | Y | UC |
| Campi et al. (2020) | Y | UC | UC | Y | Y | N | Y | Y | UC |
| Charsouei, et al. (2020) | NA | N | Y | Y | Y | Y | Y | Y | UC |
| De Joode et al (2020) | Y | UC | Y | Y | Y | N | Y | Y | NA |
| Erdem et al. (2020) | Y | UC | UC | Y | Y | N | Y | Y | UC |
| Fox et al. (2021) | N | UC | UC | Y | Y | Y | Y | Y | UC |
| Frey et al. (2020) | N | UC | UC | Y | Y | Y | Y | Y | UC |
| Frey et al. (2021) | N | UC | UC | Y | Y | Y | Y | Y | UC |
| Gebbia et al. (2020) | Y | UC | UC | Y | Y | N | Y | Y | UC |
| Gheorghe et al. (2020) | Y | Y | UC | Y | Y | N | Y | Y | Y |
| Ghosh et al. (2020) | Y | UC | UC | Y | Y | N | Y | Y | UC |
| Greco et al. (2020) | Y | UC | UC | Y | Y | Y | Y | Y | UC |
| Kim et al. (2020) | NA | N | UC | Y | Y | Y | Y | Y | Y |
| Kosir et al. (2020) | N | UC | UC | Y | Y | Y | Y | Y | UC |
| Lou et al. (2020) | N | UC | UC | Y | Y | Y | Y | Y | Y |
| Massicotte et al. (2020) | UC | UC | UC | Y | Y | Y | Y | Y | UC |
| Mitra et al (2020) | Y | Y | UC | Y | Y | N | Y | Y | Y |
| Papautsky & Hamlish et al. (2020) | N | UC | UC | Y | Y | N | Y | Y | UC |
| Pigozzi et al (2020) | Y | UC | UC | Y | Y | N | Y | Y | Y |
| Singh et al. (2020) | NA | N | UC | Y | Y | N | Y | Y | UC |
| Wang Y. et al. (2020) | Y | Y | UC | Y | Y | Y | Y | Y | Y |
| Yildirim et al. (2021) | UC | UC | UC | Y | Y | Y | Y | Y | Y |

| **II CROSS SECTIONAL ANALYTICAL STUDIES**  1. Were the criteria for inclusion in the sample clearly defined?  2. Were the study subjects and the setting described in detail?  3. Was the exposure measured in a valid and reliable way?  4. Were objective, standard criteria used for measurement of the condition?  5. Were confounding factors identified?  6. Were strategies to deal with confounding factors stated?  7. Were the outcomes measured in a valid and reliable way?  8. Was appropriate statistical analysis used? | | | | | | | | |
| --- | --- | --- | --- | --- | --- | --- | --- | --- |

| **Authors** | **Q1** | **Q2** | **Q3** | **Q4** | **Q5** | **Q6** | **Q** | **Q8** |
| --- | --- | --- | --- | --- | --- | --- | --- | --- |
| Catania et al. (2020) | Y | Y | Y | Y | Y | Y | N | Y |
| Chiax et al (2020) | Y | Y | Y | Y | Y | N | Y | Y |
| Erlan-Barak et al. (2020) | Y | Y | Y | Y | Y | N | Y | Y |
| Gultekin et al (2020) | Y | Y | Y | Y | Y | Y | Y | Y |
| Han et al. (2020) | Y | Y | Y | Y | Y | Y | Y | Y |
| Hill et al. (2021) | Y | Y | Y | Y | Y | Y | Y | Y |
| Islam et al. (2020) | Y | Y | Y | Y | Y | Y | Y | Y |
| Jeppesen et al (2020) | Y | Y | Y | Y | Y | Y | Y | Y |
| Juanjuan et al (2020) | Y | Y | Y | Y | Y | Y | Y | Y |
| Kamposioras et al. (2020) | Y | Y | Y | Y | Y | Y | Y | Y |
| Leach et al 2021 | Y | Y | Y | Y | Y | Y | N | Y |
| Miaskowski et al. (2020) | Y | Y | Y | Y | Y | Y | Y | Y |
| Ng et al. (2020) | Y | Y | Y | Y | Y | Y | Y | Y |
| Rajan et al. (2020) | Y | Y | Y | Y | Y | Y | N | Y |
| Shinan-Altman et al. (2020) | Y | Y | Y | Y | Y | Y | Y | Y |
| Sigorski et al (2020) | Y | Y | Y | Y | Y | Y | Y | Y |
| Vanni et al (2020) | Y | Y | N | UC | Y | Y | N | Y |
| Yang S et al (2020) | Y | Y | Y | Y | Y | Y | Y | Y |
| Yang g et al | Y | Y | Y | Y | Y | UC | Y | Y |

| **III QUALITATIVE STUDIES**  1. Is there congruity between the stated philosophical perspective and the research methodology?  2. Is there congruity between the research methodology and the research question or objectives?  3. Is there congruity between the research methodology and the methods used to collect data?  4. Is there congruity between the research methodology and the representation and analysis of data?  5. Is there congruity between the research methodology and the interpretation of results?  6. Is there a statement locating the researcher culturally or theoretically?  7. Is the influence of the researcher on the research, and vice- versa, addressed?  8. Are participants, and their voices, adequately represented?  9. Is the research ethical according to current criteria or, for recent studies, and is there evidence of ethical approval by an appropriate body?  10. Do the conclusions drawn in the research report flow from the analysis, or interpretation, of the data? | | | | | | | | | | |
| --- | --- | --- | --- | --- | --- | --- | --- | --- | --- | --- |

| **Authors** | **Q1** | **Q2** | **Q3** | **Q4** | **Q5** | **Q6** | **Q7** | **Q8** | **Q.9** | **Q.10** |
| --- | --- | --- | --- | --- | --- | --- | --- | --- | --- | --- |
| Chia et al. (2020) | UC | Y | Y | Y | Y | Y | Y | Y | Y | Y |
| Kosir et al. (2020) | Y | Y | Y | Y | Y | Y | Y | Y | Y | Y |
| Leach et al 2021 | Y | Y | Y | Y | Y | Y | UC | Y | UC | Y |
| Phillip et al (2020) | Y | Y | Y | Y | Y | Y | UC | Y | Y | Y |
| Souza et al (2020) | Y | Y | Y | Y | Y | N | N | Y | Y | Y |
| Yan et al. (2020) | N | N | Y | UC | UC | N | N | Y | N | Y |

| **IV Consensus on Health Economic Criteria (CHEC)-List** | | |
| --- | --- | --- |
|  | Mari et al. (2020) | Parikh et al. (2020) |
| 1. Is the study population clearly described? | Y | Y |
| 2. Are competing alternatives clearly described? | NA | Y |
| 3. Is a well-defined research question posed in answerable form? | Y | Y |
| 4. Is the economic study design appropriate to the stated objective? | Y | Y |
| 5. Is the chosen time horizon appropriate to include relevant costs and consequences? | N | Y |
| 6. Is the actual perspective chosen appropriate? | Y | Y |
| 7. Are all important and relevant costs for each alternative identified? | Y | Y |
| 8. Are all costs measured appropriately in physical units? | Y | Y |
| 9. Are costs valued appropriately? | Y | Y |
| 10. Are all important and relevant outcomes for each alternative identified? | NA | NA |
| 11. Are all outcomes measured appropriately? | NA | NA |
| 12. Are outcomes valued appropriately? | NA | NA |
| 13. Is an incremental analysis of costs and outcomes of alternatives performed? | NA | NA |
| 14. Are all future costs and outcomes discounted appropriately? | NA | NA |
| 15. Are all important variables, whose values are uncertain, appropriately subjected to sensitivity analysis? | N | Y |
| 16. Do the conclusions follow from the data reported? | Y | Y |
| 17. Does the study discuss the generalizability of the results to other settings and patient/ client groups? | N | N |
| 18. Does the article indicate that there is no potential conflict of interest of study researcher(s) and funder(s)? | Y | Y |
| 19. Are (a) ethical and (b) distributional issues discussed appropriately? | (a) Y (b) Y | (a) N (b)Y |

N = No, NA = Not applicable, UC = unclear, Y = Yes
